# High prevalence of clonal hematopoiesis‐type genomic abnormalities in cell‐free DNA in invasive gliomas after treatment

**DOI:** 10.1002/ijc.33481

**Published:** 2021-02-05

**Authors:** Ryosuke Okamura, David E. Piccioni, Amélie Boichard, Suzanna Lee, Rebecca E. Jimenez, Jason K. Sicklick, Shumei Kato, Razelle Kurzrock

**Affiliations:** ^1^ Center for Personalized Cancer Therapy UC San Diego Moores Cancer Center La Jolla California USA; ^2^ Division of Neuro‐oncology, Department of Neurosciences UC San Diego Moores Cancer Center La Jolla California USA; ^3^ Division of Surgical Oncology, Department of Surgery UC San Diego Moores Cancer Center La Jolla California USA

**Keywords:** cell‐free DNA, CH, clonal hematopoiesis, glioblastoma multiforme, glioma, liquid biopsy, molecular profiling

## Abstract

Plasma cell‐free DNA (cfDNA) is emerging as an important diagnostic tool in cancer. However, cfDNA alterations may differ from those in tissue and sometimes may reflect processes unrelated to the cancer, including clonal hematopoiesis (CH). We examined plasma cfDNA, tested by next‐generation sequencing (NGS), for characterized alterations (excluding variants of unknown significance) in 135 patients with invasive glioma. Overall, 21% (28/135) had ≥1 alteration; 17% (23/135) had CH‐type cfDNA mutations. Temozolomide (a mutagenic alkylating agent) with concurrent radiation therapy prior to blood draw was significantly associated with an increase in CH‐type mutations, even after age, race/ethnicity, and WHO‐grade were considered as confounders (odds ratio [95% confidence interval, CI] 8.98 [1.13‐71.46]; *P* = .04; multivariable analysis). Further, of 18 patients with invasive glioma who had both cfDNA and tissue DNA NGS and had ≥1 cfDNA alteration, 16 (89%) had ≥1 cfDNA alteration not found in their tissue DNA, including CH‐type alterations in genes such as *TP53* (most common), *ATM*, *GNAS*, and *JAK2*. Altogether, 87% of cfDNA alterations (20/23) observed in the 18 patients were implicated in CH. Finally, examining all 135 patients, CH‐type cfDNA mutations were an independent prognostic factor for shorter survival (hazard ratio [95% CI] 3.28 [1.28‐8.40]; *P* = .01). These findings emphasize that not all characterized cfDNA alterations detected in patients with solid tumors are cancer‐related. Importantly, in patients with invasive gliomas who have had prior temozolomide and radiation, CH‐related alterations in cfDNA are frequent and correlate with poor outcomes.

## Abstract 2

**What's new?**

Tissue biopsy for brain tumors presents significant challenges, making less‐invasive molecular profiling with plasma cell‐free DNA (cfDNA) an appealing alternative. However, whether alterations detected in cfDNA, including clonal hematopoiesis (CH), reflect processes in brain tumor tissue remains uncertain. In this investigation of CH‐associated mutations in plasma‐derived cfDNA from patients with invasive gliomas, 87 percent of characterized cfDNA alterations were implicated in CH, indicating that not all cfDNA alterations are cancer‐related. In addition, temozolomide/radiation therapy prior to blood draw for cfDNA was associated with potential CH‐type cfDNA mutation detection. Potential CH‐type cfDNA alterations were an independent predictor of shorter overall survival.

**Abbreviations**
%cfDNA: mutant allele frequency
AA: anaplastic astrocytoma
AO: anaplastic oligodendroglioma
CAP: College of American Pathologist
cfDNA: cell‐free DNA
CH: clonal hematopoiesis
CI: confidence interval
CLIA: clinical laboratory improvement amendments
DA: diffuse astrocytoma
GBM: glioblastoma multiforme
HR: hazard ratio
NGS: next‐generation sequencing
OD: oligodendroglioma
OR: odds ratio
OS: overall survival
RT: radiation therapy
TMZ: temozolomide
VUS: variant of unknown significance

## 1 INTRODUCTION

Despite advances in the therapeutics of diverse cancers, treatment options for progressive brain tumors remain limited. Glioblastoma multiforme (GBM), in particular, has a dismal prognosis, with a 10% survival at 5 years and a median survival <15 months, even after the introduction of temozolomide (TMZ).^1^ There is an unmet need to better understand the biology of these lethal cancers. Repeated tumor tissue biopsy for sequencing is difficult in brain tumors and less‐invasive molecular profiling using plasma‐derived cell‐free DNA (cfDNA) is now promising. In several tumor types, the prevalence of characterized cfDNA alterations is 60% to 80% and concordance rates between cfDNA and tissue characterized alterations have been reported to be from 50% to 90%.^2^, ^3^, ^4^, ^5^, ^6^

In respect to brain gliomas, it has been suspected that leakage of DNA from intracranial lesions into the peripheral circulation may be somewhat attenuated by the blood‐brain‐barrier and that detectable cfDNA in patients with brain tumors is lower than that in other types of tumors. However, several studies have shown that a subset of patients harbor detectable cfDNA.^7^, ^8^, ^9^ Even so, the data regarding liquid biopsy in patients with brain gliomas remain remarkably sparse.

There are many reasons that could account for differences in alterations between cfDNA and tissue: (a) tissue sequencing examines only the small piece of tissue sequenced while cfDNA may detect shed DNA that reflects tumor heterogeneity from multiple metastatic sites; (b) cfDNA may be suppressed by therapy; (c) cfDNA is present in small amounts and requires sensitive techniques; and (d) cfDNA (but also possibly tissue) may reflect detection of alterations due to clonal hematopoiesis (CH).^10^, ^11^, ^12^, ^13^ CH is a relatively newly described condition that reflects the presence of detectable hematopoietic clones in peripheral blood of individuals without hematologic malignancy. CH associated mutations are found in genes associated with myelodysplastic syndromes, acute myeloid leukemia, lymphoma, and other hematologic malignancies. Alterations in the following genes have been implicated as CH in some circumstances: *ASXL1*, *ATM*, *BCOR*, *CALR*, *CBL*, *CEBPA*, *CREBBP*, *DNMT3A*, *ETV6*, *EZH2*, *FLT3*, *GNAS*, *IDH1*, *IDH2*, *JAK2*, *KIT*, *KRAS*, *MPL*, *MYD88*, *NPM1*, *NRAS*, *PPM1D*, *RUNX1*, *SETD2*, *SF3B1*, *SH2B3*, *SRSF2*, *STAG2*, *STAT3*, *TET2*, *TP53*, *U2AF1*, *WT1*, and *ZRSR2*.^14^, ^15^ In general, the prevalence of CH mutations increases with age and is over 7% in people aged 60 years or older without a detectable solid or hematologic malignancy.^15^, ^16^, ^17^ Also, CH mutations occur more frequently in white race.^15^ In patients with cancer, certain types of environmental insults, such as exposure to chemotherapy or radiation, can increase the frequency of genomic mutations.^18^, ^19^ In fact, it has been reported that CH mutations were detected in 25% of patients with cancer (who did not have hematologic malignancies).^15^

In our study, we analyzed cfDNA samples from 135 patients with invasive gliomas, defined as diffuse astrocytic or oligodendroglial tumors, and assessed the potential impact of CH‐type mutations on cfDNA sequencing and correlation with clinical outcomes.

## 2 METHODS

### 2.1 Study population

Patients who were diagnosed with diffuse astrocytoma (DA), oligodendroglioma (OD), anaplastic oligodendroglioma (AO), anaplastic astrocytoma (AA), or GBM and who had cfDNA analysis were eligible. If a patient had multiple cfDNA analyses, the oldest report was used for analyses.

### 2.2 Clinical‐grade next‐generation sequencing

#### 2.2.1 Cell‐free DNA

All cfDNA next‐generation sequencing (NGS) was performed at a clinical laboratory improvement amendments (CLIA)‐licensed and College of American Pathologist (CAP)‐accredited clinical laboratory, *Guardant Health*, *Inc*. (http://www.guardanthealth.com). As previously reported, the cfDNA assay sequences cancer‐associated genes to identify somatic alterations with high sensitivity and high specificity (>99.9999%) (54‐73 genes: Table S1).^12^ The original panel of 54‐gene (point mutations in 54 genes and amplifications in 3 genes) was used for 45 samples; the second panel of 68‐gene (point mutations in 68 genes, amplifications in 16 genes, fusions in 4 genes, and insertions/deletions in *EGFR*) for 61 samples; the third panel of 70‐gene for 3 samples (point mutations in 70 genes, amplifications in 18 genes, fusions in 6 genes, and insertions/deletions in *EGFR* and *ERBB2*); and the most recent panel of 73‐gene (point mutations in 73 genes, amplifications in 17 genes, fusions in 6 genes, and insertions/deletions in 22 genes) for 26 samples. Among the common CH‐associated genes,^14^, ^15^ mutations in *ATM*, *GNAS*, *IDH1*, *IDH2*, *JAK2*, *KIT*, *KRAS*, *MPL*, and *NRAS* were assessed throughout these panels. We assessed characterized alterations as well as variants of unknown significance (VUSs) (including synonymous mutations in cfDNA). Mutant allele frequency (%cfDNA) was calculated as the number of mutant molecules divided by the total number of DNA fragments in each mutated gene.

#### 2.2.2 Tissue‐DNA

For comparisons with cfDNA, tissue‐DNA NGS was also used if available. Tissue samples were sequenced at a CLIA‐licensed, CAP‐accredited laboratory, *Foundation Medicine*, *Inc*. (236‐324 genes; https://www.foundationmedicine.com)^20^, ^21^ or University of California San Diego Health Clinical Laboratories (397 genes) (Table S2).Tissue samples are reviewed by a pathologist and only samples with tumor purity of >20% are sequenced.

### 2.3 Definition and statistical analysis

Diagnosis at the time of blood draw for cfDNA was used for our study. For exploration of independent prognostic factors for the overall survival (OS), we used Cox proportional hazard model in multivariate analysis to estimate hazard ratio (HR) with 95% confidence interval (CI). All variables with *P* < .1 in the univariate analyses were entered into the multivariate analysis. Statistical analysis was performed by RO using SPSS v25 software (IBM‐Corporation).

## 3 RESULTS

### 3.1 Genomic alterations in invasive glioma cfDNA

**FIGURE 1:**
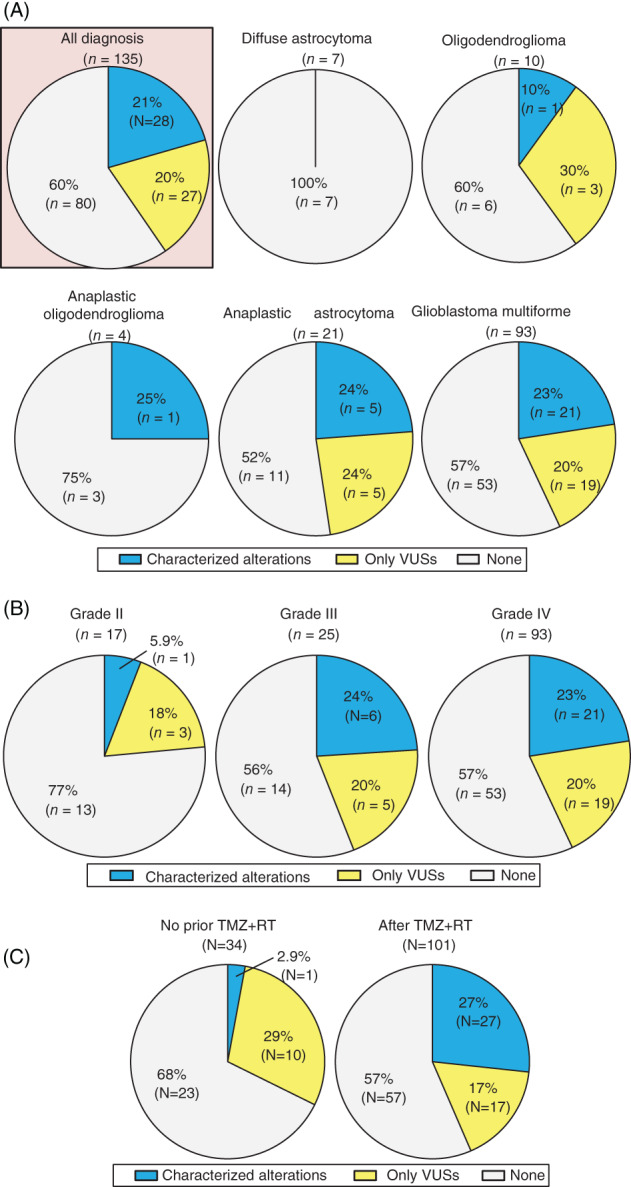
Detection rate of genomic alterations in cfDNA among patients with brain tumors (N = 135). A, According to tumor diagnosis. B, According to WHO‐grades.^35^ The rate of characterized alteration in grade III/IV vs grade II tumors was 23% vs 6% (*P* = .20). Note that grade II is sum of diffuse astrocytoma and the oligodendroglioma; grade III is sum of anaplastic oligodendroglioma and anaplastic astrocytoma. C, According to presence of temozolomide with concurrent radiotherapy (TMZ + RT) prior to blood draw for cfDNA sequencing. The rate of characterized cfDNA alteration in patients with no prior TMZ + RT vs those whose analyzed after TMZ + RT is 2.9% vs 27% (*P* = .003). cfDNA, cell‐free DNA; TMZ + RT, temozolomide with concurrent radiation therapy; VUS, variant of unknown significance; WHO, World Health Organization

Overall, 135 eligible patients with invasive gliomas had cfDNA analysis: 93, GBM patients (69%); 21, AA (16%); 10, OD (7%); 7, DA (5%); and 4, AO (3%) (Table 1). Of the 135 patients, 21% (N = 28) had ≥1 characterized alteration and an additional 20% (N = 27) had only VUSs in cfDNA. When assessed according to 2016 central nervous system WHO classification, characterized alterations were detected in 23% (N = 21/93) of GBM and 24% (N = 5/21) of AA, while only one patient with each of OD (N = 1/10, 10%) and AO (N = 1/4, 25%), and no DA patient had characterized cfDNA alterations (Figure 1A).

**TABLE 1:** Characteristics of patients with brain gliomas (N = 135)

Characteristics (N = 135)	N (%)
**Median age (range) (y)** ^a^	55.8 (22.5‐86.8)
**Gender**	
Men	88 (65.2%)
Women	47 (34.8%)
**Race/ethnicity**	
White (non‐Hispanic)	94 (69.6%)
Hispanic	18 (13.3%)
Asian	10 (7.4%)
African American	1 (0.7%)
Other/unknown	12 (8.9%)
**Types of brain tumors**	
*Diffuse astrocytoma (grade II)*	7 (5.2%)
*IDH* wild type	3 (2.2%)
*IDH* mutant	3 (2.2%)
NOS	1 (0.7%)
*Oligodendroglioma (grade II)*	10 (7.4%)
*IDH* mutant and 1p/19q‐codeleted	8 (5.9%)
NOS	2 (1.5%)
*Anaplastic oligodendroglioma (grade III)*	4 (3.0%)
*IDH* mutant and 1p/19q‐codeleted	2 (1.5%)
NOS	2 (1.5%)
*Anaplastic astrocytoma (grade III)*	21 (16%)
*IDH* wild type	7 (5.2%)
*IDH* mutant	12 (8.9%)
NOS	2 (1.5%)
*Glioblastoma multiforme (grade IV)*	93 (69%)
*IDH* wild type	79 (59%)
*IDH* mutant	7 (5.2%)
NOS	7 (5.2%)

Abbreviations: %cfDNA, mutant allele frequency; cfDNA, cell‐free DNA; IQR, interquartile range; NOS, not otherwise specified; VUS, variant of unknown significance.

^a^ Age at the time of blood draw for cfDNA analysis.

**FIGURE 2:**
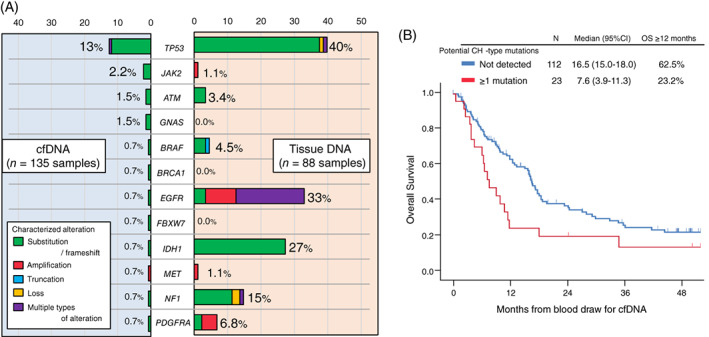
A, Detection rate (percentage of patients) of characterized genomic alterations among patients with brain gliomas. cfDNA analysis was performed in 135 patients while tissue‐DNA was performed in 88 patients. Characterized cfDNA alterations occurred in these 12 genes. The loci altered may not be the same in the cfDNA and tissue, even if the gene is the same (Table S3). B, Kaplan‐Meier curve for overall survival (OS) from cfDNA blood test depending on the presence of potential CH‐type mutations in cfDNA among patients with brain gliomas (N = 135). cfDNA, cell‐free DNA; CH, clonal hematopoiesis; CI, confidence interval; HR, hazard ratio; OS, overall survival

When categorized by tumor grade, characterized cfDNA alterations were detected in 23% (N = 21/93) and 24% (N = 6/25) of grade IV and III tumors, respectively, while only 6% (N = 1/17) of grade II tumors had characterized alterations (grade IV/III vs grade II, 23% vs 6%, *P* = .20) (Figure 1B). Characterized cfDNA alterations occurred in 12 different genes, the most common gene altered in cfDNA being *TP53* (13%, N = 17), followed by *JAK2* (2.2%, N = 3), *ATM* (1.5%, N = 2), and *GNAS* (1.5%, N = 2) (Figure 2A).

### 3.2 Characterized tissue DNA alterations

Among the 135 patients with glioma, tissue DNA analysis was available in 88 patients (65%). Median number of characterized tissue DNA alterations was 5 (range = 0‐12) and 99% of the patients (N = 87/88) had at least one characterized alteration. A total of 89 different genes were involved and the most common genes altered in tissue DNA were *CDKN2A/B* (43%, N = 38), *TERT* (43%, N = 38), and *TP53* (40%, N = 35) (Figure S1).

### 3.3 Comparison of cfDNA and tissue DNA to identify CH mutations

When comparing characterized alterations in the 12 genes altered (*ATM*, *BRAF*, *BRCA1*, *EGFR*, *FBXW7*, *GNAS*, *IDH1*, *JAK2*, *MET*, *NF1*, *PDGFRA*, and *TP53*) in cfDNA with those in tissue DNA, the detection rate of characterized alterations for each gene was mostly lower in cfDNA than in tissue DNA (Figure 2A). For instance, 40% of patients had a characterized *TP53* alteration in their tissue DNA while only 13% of patients had a characterized *TP53* alteration in their cfDNA. In this series, 88 patients with invasive glioma had both cfDNA and tissue DNA NGS performed. Among them, 18 patients had at least one characterized cfDNA alteration. Of the 18 patients, only two patients had ≥1 identical characterized alteration in cfDNA and tissue DNA (ID#105, *IDH1* R132H; and ID#120, *EGFR* A289V), and, overall, 17 (all except for ID#105) had ≥1 characterized cfDNA alteration not found in their tissue DNA (Table S3). The cfDNA alterations not found in tissue included *ATM*, *GNAS*, *JAK2*, *PDGFRA*, and *TP53*. Mutations in all these genes except *PDGFRA* have been potentially associated with CH.^15^, ^22^ Therefore, 16 of the 18 patients (except ID#50 and #105) had ≥1 cfDNA alterations that have been implicated in CH. Altogether, 18% (16/88) of the patients whose tissue DNA NGS were also available had ≥1 CH‐type cfDNA alteration. The most common potential CH‐type alteration was in *TP53* (11 patients had *TP53* cfDNA mutations) (Table S3). Of potential interest, 17 of the 18 patients (except ID#114) had received temozolomide plus radiotherapy therapy prior to blood draw for cfDNA.

In 10 patients who had characterized cfDNA alterations and did not have tissue DNA NGS performed, potential CH‐type mutations were observed in seven patients (70%) (*MET* amplification and alterations in *BRAF*, *BRCA1*, *FBXW7*, and *NF1* were not considered as CH) (Table S3). Altogether potential CH‐type mutations were observed in 23 of 135 glioma patients; of patients with ≥1 characterized alteration, 82% (23/28) had CH‐type mutations. Median variant allele fraction for the potential CH‐type mutations was 0.3% (range = 0.1%‐4.2%).

### 3.4 The percent of patients with CH‐type mutations increased after therapy with temozolomide and radiation

In this series, 101 of 135 patients (75%) received temozolomide with concurrent radiation therapy (TMZ + RT) prior to blood draw for cfDNA. Patients who received TMZ alone (N = 5) or radiation alone (N = 5) prior to blood draw for cfDNA were treated as those “without TMZ + RT” in the analyses. Detection rate of characterized cfDNA alterations was significantly higher in blood samples biopsied after TMZ + RT than those without prior TMZ + RT (27% [27/101] vs 2.9% [1/34], *P* = .003) (Figure 1C). Moreover, when only potential CH‐type mutations were considered, the frequency was higher in blood samples biopsied after TMZ + RT than those without prior TMZ + RT (22% [22/101] vs 2.9% [1/34], *P* = .009). The multivariate analysis confirmed the significant independent association of potential CH‐type mutations with prior TMZ + RT therapy even after age, race/ethnicity, and WHO‐grade were considered as confounders (odds ratio [OR] [95% CI] 8.98 [1.13‐71.46]; *P* = .04) (Table 2).

**TABLE 2:** Multivariate analysis for association of prior treatment with CH‐type mutations in glioma patients (N = 135)

		Multivariate analysis	
Patient background	Frequency of *Potential CH* (%)	OR (95% CI)	*P* value^a^
*Age* ^b^			
≥56 y (N = 67) vs <56 y (N = 68)	19% vs 15%	1.15 (0.44‐3.05)	.77
*Race/ethnicity*			
White (non‐Hispanic) (N = 94) vs others (N = 41)	22% vs 4.9%	5.94 (1.27‐27.71)	**.007**
*WHO grade*			
Grade IV/III (N = 118) vs grade II (N = 17)	19% vs 5.9%	2.09 (0.23‐18.56)	.51
*Treatment prior to blood draw*			
Temozolomide and radiation (N = 101) vs not (N = 34)	22% vs 2.9%	8.98 (1.13‐71.46)	**.04**

Abbreviations: cfDNA, cell‐free DNA; CH, clonal hematopoiesis; CI, confidence interval; OR, odds ratio; WHO, World Health Organization.

^a^ The bold values represent the statistically significant *P*‐values (<.05).

^b^ At the time of blood draw for cfDNA (years), age 56 years is the median and was chosen as the cutoff; other cutoffs, for example, 65 or 70 years, were also not significant (*P* = .54 and *P* = .69, respectively).

### 3.5 CH‐type alterations in cfDNA correlated with shorter OS

When all 135 patients were considered, the detection of potential CH‐type cfDNA mutations was significantly associated with shorter OS time (median [12‐month rate], 7.6 months vs 16.5 months [23% vs 63%]; *P* = .02) (Figure 2B). Among 135 patients, the 28 patients who had ≥1 characterized cfDNA alteration (including CH or non‐CH) showed a tendency toward shorter median OS time than those with no characterized cfDNA alterations detected (median [12‐month rate], 9.1 vs 16.6 [30% vs 63%]; *P* = .08) (Figure S2 and Table S4). Even when age (≥56 vs <56 years), gender, race/ethnicity, *IDH*‐status, WHO‐grade, total mutant allele frequency (%cfDNA), and cfDNA *TP53* alterations were considered as possible confounding factors, the multivariate analysis showed that the presence of potential CH‐type cfDNA mutations was significantly and independently associated with shorter OS time (HR [95% CI] 3.28 [1.28‐8.40]; *P* = .01) (Table 3). *IDH*‐wild type (or not otherwise specified) (HR [95% CI] 3.54 [1.72‐7.29]; *P* = .001) and WHO‐grade IV vs II or III (HR [95% CI] 2.39 [1.32‐4.34]; *P* = 0.004) were also independently associated with shorter OS time in the multivariate analysis.

**TABLE 3:** Multivariate analysis for factors associated with overall survival from date of cfDNA analysis in patients with brain tumors (N = 135)

Characteristics	Univariate analysis^a^		Multivariate analysis	
	Median OS months	*P* value^b^	HR (95% CI)	*P* value^b^
*Age* ^c^				
≥56 y (N = 67) vs <56 y (N = 68)	11.9 vs 16.5	**.02**	0.87 (0.56‐1.36)	.55
*Gender*				
Men (N = 88) vs women (N = 47)	15.7 vs 17.2	.49	—	—
*Race/ethnicity*				
White (non‐Hispanic) (N = 94) vs others (N = 41)	15.7 vs 16.6	.60	—	—
*Types of tumor*				
*IDH* wild type/NOS (N = 103) vs *IDH* mutant^d^ (N = 32)	11.5 vs NR	<**.001**	3.54 (1.72–7.29)	**.001**
WHO grade IV (N = 93) vs grade II/III (N = 42)	11.3 vs NR	<**.001**	2.39 (1.32‐4.34)	**.004**
*Alteration detected in cfDNA*				
≥1 potential CH‐type mutations (N = 23) vs none (N = 112)	7.6 vs 16.5	**.02**	3.28 (1.28–8.40)	**.01**
Total %cfDNA^e^ ≥0.3% (N = 21) vs <0.3% (N = 114)	9.1 vs 16.3	.40	—	—
Characterized *TP53* alteration (N = 17) vs not (N = 118)	7.2 vs 16.3	.08	0.39 (0.14‐1.13)	.08

Abbreviations: %cfDNA, mutant allele frequency; cfDNA, cell‐free DNA; CH, clonal hematopoiesis; CI, confidence interval; HR, hazard ratio; NOS, not otherwise specified; NR, not reached to 50%; OS, overall survival; VUS, variant of unknown significance.

^a^ Factors with *P* value <.10 in univariate analysis were included in the multivariate analysis.

^b^ The bold values represent the statistically significant p‐values (<0.05).

^c^ Age 56 years is the median. Examining age as a continuous variable was also not significant (*P* = .21).

^d^ Generally *IDH1* mutation.

^e^ All types of alterations (characterized alteration, VUS, and synonymous mutation) were considered. Dichotomized at the 75 percentile of 0.3%.

## 4 DISCUSSION

Along with the increased use of plasma‐derived cfDNA analysis as a diagnostic tool or for detecting therapeutic biomarkers among advance diverse cancers, the specificity (false positive) of cfDNA NGS reports is becoming an important issue to be discussed. “False positive” cfDNA alterations may reflect alterations not present in the tumor, but rather CH‐type mutations, which are associated with blood cells derived from a hematopoietic stem cell and often detected even in people without any hematologic malignancies.^14^, ^15^, ^16^, ^17^ In general, characterized cfDNA alterations are often considered targetable by anticancer drugs in the precision therapeutic approaches for patients with solid tumors, but CH‐type mutations should be removed from the therapeutic equation.

In this series, 21% of 135 glioma patients have at least one characterized alteration in peripheral blood‐derived plasma cfDNA NGS (Figure 1A). The rate was consistent with the 10% to 29% described in previous reports.^8^, ^23^ The subset of patients with detectable characterized cfDNA increased with WHO‐grade: 23% to 24% in grade III and IV (AO, AA, or GBM) vs 6% in grade II (DA or OD) (*P* = .20), albeit not statistically significant (Figure 1B). The characterized cfDNA alterations were seen in the 12 genes listed in Figure 2A, and the most common alterations in cfDNA were in *TP53* (13%) followed by *JAK2* (2.2%), *ATM* (1.5%), and *GNAS* (1.5%). These findings were different from tissue DNA NGS because allelic loss or deletion in *CDKN2A*/*B* (43%) and mutations or amplifications in *TERT* (43%) were the most common characterized alterations detected in tissue DNA (Figure S1). Interestingly, among the 12 genes involved in the characterized cfDNA alterations, *BRCA1*, *FBXW7*, and *GNAS* genes were not altered in 88 samples for tissue DNA. Also, tissue DNA alterations in each of *JAK2* and *MET* were seen in only one case. Thus, there were large discrepancies in results between clinical‐grade cfDNA and tissue DNA NGS for invasive glioma patients.

In the patients who had both cfDNA and tissue DNA NGS performed and whose cfDNA showed ≥1 characterized alteration, characterized cfDNA alterations not found in tissue DNA could be seen in 94% of patients (Table S3). These cfDNA alterations were comprised of *ATM*, *GNAS*, *JAK2*, *PDGFRA*, and *TP53* mutations. We considered that the mutations in *ATM*, *GNAS*, *JAK2*, and *TP53* genes (but not the *PDGFRA* mutation in ID#50) were possibly CH‐type mutations (Table S3). Interestingly, the common cfDNA alterations in patients with invasive glioma were mostly implicated in CH. In general, *ASXL1*, *DNMT3A*, *PPM1D*, and *TET2* genes are commonly involved in CH, but these were not included in the sequencing cfDNA panels of the current study. Also, a previous study suggested that not all CH variants can be detected in the blood cell‐derived DNA.^24^ Although it is difficult to determine if these mutations are CH or cancer‐related, over 80% of the invasive glioma patients with characterized cfDNA alterations detected had at least one potential CH‐type mutations in our analysis.

We also observed that the prevalence of characterized cfDNA alterations in patients with invasive gliomas was significantly higher in blood samples biopsied after TMZ + RT (27% vs 2.9%, *P* = .003) (Figure 1C). This finding remained consistent among the subset of 93 patients with GBM (28% [21/74] vs 0% [0/19], *P* = .005) (Figure S3). Notably, TMZ + RT was highly associated with the increase of potential CH‐type mutations (OR [95% CI] 8.98 [1.13‐71.46]; *P* = .04) even after age, race/ethnicity, and WHO‐grade were considered as confounding factors (Table 2). TMZ + RT therapy have been used as the standard treatment for invasive gliomas and both have been reported as mutagenic, including for bone marrow.^15^, ^25^, ^26^ TMZ, an alkylating pro‐drug, methylates DNA and leads to genomic instability and eventually cancer cell death, but also causes inactivating mutation in the mismatch repair genes, inducing hypermutations.^27^, ^28^ Similarly, radiation mutagenesis in which free radicals affect the cytoplasm and lead to DNA damage is well known.^29^ In this series, *MET* amplification and mutations in *PDGFRA*, *EGFR*, *BRAF*, *BRCA1*, *FBXW7*, and *NF1* were considered as non‐CH type alterations. Although cfDNA *IDH1* mutations generally can be considered as potential CH‐type, an *IDH1* mutation in cfDNA of patient ID#105 was considered as a non‐CH type alteration because her tissue DNA also showed the identical *IDH1* alteration (Table S3). Thus, only 6% (8/135) of the patients with invasive glioma had non‐CH type characterized cfDNA alterations which may be informative for treatment decisions (Table S3).

Previous studies reported that CH is associated with increased all‐cause death and shorter OS time, suggesting that the increased death was caused by cardiovascular or ischemic stroke events rather than hematologic malignancies.^16^, ^30^, ^31^ Our study also demonstrated that the presence of CH‐type mutations in cfDNA was independently associated with worse OS in patients with invasive gliomas (HR [95% CI] 3.28 [1.28‐8.40], *P* = .01 [multivariate]) (Table 3).

There were several limitations associated with the study. First, some other studies defined a CH mutation by comparing its %cfDNA with the allele frequency of the buffy coat‐derived DNA or tumor tissue DNA.^15^, ^24^, ^32^ However, we did not assess leukocytes. Also, we failed to capture some of the genomic alterations that were commonly involved in CH (the cfDNA panels did not include *ASXL1*, *DNMT3A*, *PPM1D*, *TET2*, etc.) (Table S1). Second, not all patients had both plasma‐derived ctDNA and tumor tissue DNA tests; therefore, future analysis comparing results in both tests should be performed with larger numbers of patients. Third, analysis of the independent influence of temozolomide and radiation was not feasible in our study due to small numbers of patients with only one of these modalities. Fourth, previous studies reported that CH mutations can also be detected in tumor tissue sequencing.^33^, ^34^ However, our study did not evaluate potential CH mutations in tissue DNA because of the lack of data regarding the variant allele frequency of tissue DNA alterations. Despite these limitations, our report suggests a high rate of CH alterations in cfDNA derived from patients with invasive glioma treated with temozolomide (a mutagenic alkylating agent) and radiation. The frequent presence of these CH‐type alterations indicates that caution is needed when interpreting the results of cfDNA in patients with invasive glioma, as many of the alterations may not be derived from the tumor. Furthermore, patients carrying these CH‐type cfDNA alterations have a shorter survival. Additional investigations are required to explain this sequence.

## CONFLICT OF INTEREST

D. E. P. is a consultant for Tocagen. J. K. S. has the following disclosure information: Research funding (Novartis Pharmaceuticals, Amgen Pharmaceuticals, and Foundation Medicine); Consultant fee (Grand Rounds [2015‐2019], Loxo Oncology [2017‐2018], Deciphera [2019], and Roche [2019]). S. K. serves as a consultant for Foundation Medicine, Inc. He receives speaker's fee from Roche and advisory board for Pfizer. He has research funding from ACT Genomics, Sysmex, Konica Minolta, and OmniSeq. R. K. receives research funding from Genentech, Merck Serono, Pfizer, Boerhringer Ingelheim, TopAlliance, Takeda, Incyte, DeBiopharm, Medimmune, Sequenom, Foundation Medicine, Konica Minolta, Grifols, OmniSeq, and Guardant, as well as consultant and/or speaker fees and/or advisory board for X‐Biotech, NeoMed, Pfizer, Actuate Therapeutics, Roche, Turning Point Therapeutics, TD2/Volastra, Bicara Therapeutics, Inc., has an equity interest in IDbyDNA and CureMatch, Inc., serves on the board of CureMatch and CureMetrix, and is a cofounder of CureMatch. The other authors have no conflicts of interest.

## ETHICS STATEMENT

This study was performed in accordance with the guidelines of the Internal Review Board‐approved/declaration of Helsinki compliant study—UCSD‐PREDICT (NCT02478931) and any investigational therapies for which the patient gave consent.

## Supporting information


**Appendix S1:** Supplementary Information

## Data Availability

The data that support the findings of our study are available from the corresponding author upon reasonable request.
