# Supplementary material for: High prevalence of clonal hematopoiesis-type genomic abnormalities in cell-free DNA in invasive gliomas after treatment
Source: Int J Cancer. Author manuscript; Available in PMC 2023 May 23. (PMC8048515; doi:10.1002/ijc.33481)

**SUPPLEMENTARY MATERIALS**

**High Prevalence of Clonal Hematopoiesis-type Genomic Abnormalities**

**in Cell-free DNA in Invasive Gliomas After Treatment**

Ryosuke Okamura^1^*, David E. Piccioni^1, 2^*, Amélie Boichard^1^, Suzanna Lee^1^, Rebecca E. Jimenez^1^,

Jason K. Sicklick^1, 3^, Shumei Kato^1^, and Razelle Kurzrock^1^

**CONTENTS**

**Supplementary Tables**

- **Supplementary Table S1.** Next-generation sequencing gene panels for blood-derived cfDNA (*Guardant, Inc*.) (N=135).
- **Supplementary Table S2**. Next-generation sequencing gene panels for tissue-DNA (N=88).
- **Supplementary Table S3.** Detailed information of invasive glioma patients harboring characterized cfDNA alterations (N=28).
- **Supplementary Table S4.** Presence of characterized cfDNA alterations and overall survival time from blood draw for cfDNA in invasive glioma patients (N=135).

**Supplementary Figures**

- **Supplementary Figure S1**. Frequency (% of patients) of genomic alterations in tissue-DNA NGS among glioma patients (N=88). Only characterized alterations are shown. A total of 89 different genes were involved (only genes altered in ≥2 samples are shown).
- **Supplementary Figure S2**. Kaplan-Meier curve for overall survival (OS) from cfDNA blood test depending on the number of characterized cfDNA alterations amongst patients with brain gliomas (N=135).
- **Supplementary Figure S3**. Detection rate of characterized cfDNA alterations among GBM patients according to presence of tumor diagnosis (N=93).

**Supplementary Table S1.** Next-generation sequencing gene panels for blood-derived cfDNA (*Guardant, Inc*.) (N=135).

**Panel S1A.** 54 gene panel (N=45 samples).

| **POINT MUTATIONS** | | | | **AMPLIFICATIONS** |
| --- | --- | --- | --- | --- |
| *ABL1* | *AKT1* | ***ALK*** | ***APC*** | *EGFR* |
| ***AR*** | *ATM* | ***BRAF*** | ***CDKN2A*** | *ERBB2* |
| *CDH1* | *CSF1R* | *CTNNB1* | ***EGFR*** | *MET* |
| ***ERBB2*** | *ERBB4* | *EZH2* | ***FBXW7*** |  |
| *FGFR1* | *FGFR2* | *FGFR3* | *FLT3* |  |
| *GNA11* | *GNAQ* | *GNAS* | *HNF1A* |  |
| *HRAS* | *IDH1* | *IDH2* | *JAK2* |  |
| *JAK3* | *KDR* | *KIT* | ***KRAS*** |  |
| ***MET*** | *MLH1* | *MPL* | ***MYC*** |  |
| ***NOTCH1*** | *NPM1* | ***NRAS*** | *PDGFRA* |  |
| ***PIK3CA*** | ***PTEN*** | *PTPN11* | ***PROC*** |  |
| ***RB1*** | *RET* | *SMAD4* | *SMARCB1* |  |
| *SMO* | *SRC* | *STK11* | *TERT* |  |
| ***TP53*** | *VHL* |  |  |  |

All exons were sequenced in genes in ***bold***.

**Panel S1B.** 68 gene panel (N=61 samples).

| **POINT MUTATIONS** | | | | **AMPLIFICATIONS** | **FUSIONS** | **INDELS** |
| --- | --- | --- | --- | --- | --- | --- |
| *AKT1* | *ALK* | ***APC*** | ***AR*** | *AR* | *ALK* | *EGFR* exon 19 deletions |
| *ARAF* | ***ARID1A*** | *ATM* | ***BRAF*** | *BRAF* | *NTRK1* | *EGFR* exon 20 insertions |
| ***BRCA1*** | ***BRCA2*** | ***CCDN1*** | ***CCDN2*** | *CCNE1* | *RET* |  |
| ***CCNE1*** | *CDH1* | ***CDK4*** | ***CDK6*** | *CDK4* | *ROS1* |  |
| ***CDKN2A*** | ***CDKN2B*** | *CTNNB1* | ***EGFR*** | *CDK6* |  |  |
| ***ERBB2*** | *ESR1* | *EZH2* | *FBXW7* | *EGFR* |  |  |
| ***FGFR1*** | ***FGFR2*** | *FGFR3* | *GATA3* | *ERBB2* |  |  |
| *GNA11* | *GNAQ* | *GNAS* | *HNF1A* | *FGFR1* |  |  |
| ***HRAS*** | *IDH1* | *IDH2* | *JAK2* | *FGFR2* |  |  |
| *JAK3* | ***KIT*** | ***KRAS*** | *MAP2K1* | *KIT* |  |  |
| *MAP2K2* | ***MET*** | *MLH1* | *MPL* | *KRAS* |  |  |
| ***MYC*** | ***NF1*** | *NFE2L2* | *NOTCH1* | *MET* |  |  |
| *NPM1* | ***NRAS*** | *NTRK1* | ***PDGFRA*** | *MYC* |  |  |
| ***PIK3CA*** | ***PTEN*** | *PTPN11* | ***RAF1*** | *PDGFRA* |  |  |
| *RET* | *RHEB* | *RHOA* | *RIT1* | *PIK3CA* |  |  |
| *ROS1* | *SMAD4* | *SMO* | *SRC* | *RAF1* |  |  |
| *STK11* | *TERT* | ***TP53*** | *VHL* |  |  |  |

Complete exon coverage for genes in ***bold***.

**Panel S1C.** 70 gene panel (N=3 samples).

| **POINT MUTATIONS** | | | | **AMPLIFICATIONS** | **FUSIONS** | **INDELS** |
| --- | --- | --- | --- | --- | --- | --- |
| *AKT1* | *ALK** | ***APC*** | ***AR*** | *AR* | *ALK* | *EGFR* exon 19 deletions |
| *ARAF* | ***ARID1A*** | *ATM* | ***BRAF*** | *BRAF* | *FGFR2* | *EGFR* exon 20 insertions |
| ***BRCA1*** | ***BRCA2*** | ***CCND1*** | ***CCND2*** | *CCND1* | *FGFR3* | *ERBB2* exon 19 deletions |
| ***CCNE1*** | *CDH1* | ***CDK4*** | ***CDK6*** | *CCND2* | *NTRK1* | *ERBB2* exon 20 insertions |
| ***CDKN2A*** | ***CDKN2B*** | *CTNNB1* | ***EGFR*** | *CCNE1* | *RET* |  |
| ***ERBB2*** | *ESR1* | *EZH2* | *FBXW7* | *CDK4* | *ROS1* |  |
| ***FGFR1*** | ***FGFR2**** | *FGFR3** | *GATA3* | *CDK6* |  |  |
| *GNA11* | *GNAQ* | *GNAS* | *HNF1A* | *EGFR* |  |  |
| ***HRAS*** | *IDH1* | *IDH2* | *JAK2* | *ERBB2* |  |  |
| *JAK3* | ***KIT*** | ***KRAS*** | *MAP2K1* | *FGFR1* |  |  |
| *MAP2K2* | ***MET*** | *MLH1* | *MPL* | *FGFR2* |  |  |
| ***MYC*** | ***NF1*** | *NFE2L2* | *NOTCH1* | *KIT* |  |  |
| *NPM1* | ***NRAS*** | *NTRK1** | ***PDGFRA*** | *KRAS* |  |  |
| ***PIK3CA*** | ***PTEN*** | *PTPN11* | ***RAF1*** | *MET* |  |  |
| ***RB1*** | *RET** | *RHEB* | *RHOA* | *MYC* |  |  |
| *RIT1* | *ROS1** | *SMAD4* | *SMO* | *PDGFRA* |  |  |
| *SRC* | *STK11* | *TERT* | ***TP53*** | *PIK3CA* |  |  |
| *TSC1* | *VHL* |  |  | *RAF1* |  |  |

Complete exon and partial intron coverage for genes in ***bold***. *Genes with asterisk include rearrangements. *MET* includes exon 14 skipping.

**Panel S1D.** 73 gene panel (N=26 samples).

| **POINT MUTATIONS** | | | | **AMPLIFICATIONS** | **FUSIONS** | **INDELS** | |
| --- | --- | --- | --- | --- | --- | --- | --- |
| *AKT1* | *ALK* | *APC* | *AR* | *AR* | *ALK* | *APC* | *ARID1A* |
| *ARAF* | *ARID1A* | *ATM* | *BRAF* | *BRAF* | *FGFR2* | *ATM* | *BRCA1* |
| *BRCA1* | *BRCA2* | *CCND1* | *CCND2* | *CCND1* | *FGFR3* | *BRCA2* | *CDH1* |
| *CCNE1* | *CDH1* | *CDK4* | *CDK6* | *CCNE1* | *NTRK1* | *CDKN2A* | *EGFR* |
| *CDKN2A* | *CTNNB1* | *DDR2* | *EGFR* | *CDK4* | *RET* | *GATA3* | *KIT* |
| *ERBB2* | *ESR1* | *EZH2* | *FBXW7* | *CDK6* | *ROS1* | *MET* | *MLH1* |
| *FGFR1* | *FGFR2* | *FGFR3* | *GATA3* | *EGFR* |  | *MTOR* | *NF1* |
| *GNA11* | *GNAQ* | *GNAS* | *HNF1A* | *ERBB2* |  | *PDGFRA* | *PTEN* |
| *HRAS* | *IDH1* | *IDH2* | *JAK2* | *FGFR1* |  | *RB1* | *SMAD4* |
| *JAK3* | *KIT* | *KRAS* | *MAP2K1* | *FGFR2* |  | *STK11* | *TP53* |
| *MAP2K2* | *MAPK1* | *MAPK3* | *MET* | *KIT* |  | *TSC1* | *VHL* |
| *MLH1* | *MPL* | *MTOR* | *MYC* | *KRAS* |  |  |  |
| *NF1* | *NFE2L2* | *NOTCH1* | *NPM1* | *MET* |  |  |  |
| *NRAS* | *NTRK1* | *NTRK3* | *PDGFRA* | *MYC* |  |  |  |
| *PIK3CA* | *PTEN* | *PTPN11* | *RAF1* | *PDGFRA* |  |  |  |
| *RB1* | *RET* | *RHEB* | *RHOA* | *PIK3CA* |  |  |  |
| *RIT1* | *ROS1* | *SMAD4* | *SMO* | *RAF1* |  |  |  |
| *STK11* | *TERT* | *TP53* | *TSC1* |  |  |  |  |
| *VHL* |  |  |  |  |  |  |  |

All clinically relevant exons for 73 genes are sequenced. *TERT* includes alterations in the promoter region. *MET* includes exon 14 skipping.

**Supplementary Table S2**. Next-generation sequencing gene panels for tissue-DNA (N=88).

**Panel S2A**. 236 gene panel of tissue-DNA (*Foundation Medicine*) (N=22 tissue samples).

| **SUBSTITUTIONS, INSERTION/DELETIONS, AND COPY NUMBER ALTERATIONS** | | | | | | | | |
| --- | --- | --- | --- | --- | --- | --- | --- | --- |
| *ABL1* | *AKT1* | *AKT2* | *AKT3* | *ALK* | *APC* | *AR* | *ARAF* | *ARFRP1* |
| *ARID1A* | *ARID2* | *ASXL1* | *ATM* | *ATR* | *ATRX* | *AURKA* | *AURKB* | *AXL* |
| *BAP1* | *BARD1* | *BCL2* | *BCL2L2* | *BCL6* | *BCOR* | *BCORL1* | *BLM* | *BRAF* |
| *BRCA1* | *BRCA2* | *BRIP1* | *BTK* | *C11orf30* | *C17orf39* | *CARD11* | *CBFB* | *CBL* |
| *CCND1* | *CCND2* | *CCND3* | *CCNE1* | *CD79A* | *CD79B* | *CDC73* | *CDH1* | *CDK12* |
| *CDK4* | *CDK6* | *CDK8* | *CDKN1B* | *CDKN2A* | *CDKN2B* | *CDKN2C* | *CEBPA* | *CHEK1* |
| *CHEK2* | *CIC* | *CREBBP* | *CRKL* | *CRLF2* | *CSF1R* | *CTCF* | *CTNNA1* | *CTNNB1* |
| *DAXX* | *DDR2* | *DNMT3A* | *DOT1L* | *EGFR* | *EP300* | *EPHA3* | *EPHA5* | *EPHB1* |
| *ERBB2* | *ERBB3* | *ERBB4* | *ERG* | *ESR1* | *EZH2* | *FAM123B* | *FAM46C* | *FANCA* |
| *FANCC* | *FANCD2* | *FANCE* | *FANCF* | *FANCG* | *FANCL* | *FBXW7* | *FGF10* | *FGF14* |
| *FGF19* | *FGF23* | *FGF3* | *FGF4* | *FGF6* | *FGFR1* | *FGFR2* | *FGFR3* | *FGFR4* |
| *FLT1* | *FLT3* | *FLT4* | *FOXL2* | *GATA1* | *GATA2* | *GATA3* | *GNA11* | *GNA13* |
| *GNAQ* | *GNAS* | *GPR124* | *GRIN2A* | *GSK3B* | *HGF* | *HRAS* | *IDH1* | *IDH2* |
| *IGF1R* | *IKBKE* | *IKZF1* | *IL7R* | *INHBA* | *IRF4* | *IRS2* | *JAK1* | *JAK2* |
| *JAK3* | *JUN* | *KAT6A* | *KDM5A* | *KDM5C* | *KDM6A* | *KDR* | *KEAP1* | *KIT* |
| *KLHL6* | *KRAS* | *LRP1B* | *MAP2K1* | *MAP2K2* | *MAP2K4* | *MAP3K1* | *MCL1* | *MDM2* |
| *MDM4* | *MED12* | *MEF2B* | *MEN1* | *MET* | *MITF* | *MLH1* | *MLL* | *MLL2* |
| *MPL* | *MRE11A* | *MSH2* | *MSH6* | *MTOR* | *MUTYH* | *MYC* | *MYCL1* | *MYCN* |
| *MYD88* | *NF1* | *NF2* | *NFE2L2* | *NFKBIA* | *NKX2-1* | *NOTCH1* | *NOTCH2* | *NPM1* |
| *NRAS* | *NTRK1* | *NTRK2* | *NTRK3* | *NUP93* | *PAK3* | *PALB2* | *PAX5* | *PBRM1* |
| *PDGFRA* | *PDGFRB* | *PDK1* | *PIK3CA* | *PIK3CG* | *PIK3R1* | *PIK3R2* | *PPP2R1A* | *PRDM1* |
| *PRKAR1A* | *PRKDC* | *PTCH1* | *PTEN* | *PTPN11* | *RAD50* | *RAD51* | *RAF1* | *RARA* |
| *RB1* | *RET* | *RICTOR* | *RNF43* | *RPTOR* | *RUNX1* | *SETD2* | *SF3B1* | *SMAD2* |
| *SMAD4* | *SMARCA4* | *SMARCB1* | *SMO* | *SOCS1* | *SOX10* | *SOX2* | *SPEN* | *SPOP* |
| *SRC* | *STAG2* | *STAT4* | *STK11* | *SUFU* | *TET2* | *TGFBR2* | *TNFAIP3* | *TNFRSF14* |
| *TOP1* | *TP53* | *TSC1* | *TSC2* | *TSHR* | *VHL* | *WISP3* | *WT1* | *XPO1* |
| *ZNF217* | *ZNF703* |  |  |  |  |  |  |  |
| **REARRANGEMENTS** | | | | | | | | |
| *ALK* | *BCL2* | *BCR* | *BRAF* | *EGFR* | *ETV1* | *ETV4* | *ETV5* | *ETV6* |
| *EWSR1* | *MLL* | *MYC* | *NTRK1* | *PDGFRA* | *RAF1* | *RARA* | *RET* | *ROS1* |
| *TMPRSS2* |  |  |  |  |  |  |  |  |

**Panel S2B**. 315 gene panel of tissue-DNA (*Foundation Medicine, Inc*) (N=52 tissue samples).

| **SUBSTITUTIONS, INSERTION/DELETIONS, AND COPY NUMBER ALTERATIONS** | | | | | | | | |
| --- | --- | --- | --- | --- | --- | --- | --- | --- |
| *ABL1* | *ABL2* | *ACVR1B* | *AKT1* | *AKT2* | *AKT3* | *ALK* | *AMER1* | *APC* |
| *AR* | *ARAF* | *ARFRP1* | *ARID1A* | *ARID1B* | *ARID2* | *ASXL1* | *ATM* | *ATR* |
| *ATRX* | *AURKA* | *AURKB* | *AXIN1* | *AXL* | *BAP1* | *BARD1* | *BCL2* | *BCL2L1* |
| *BCL2L2* | *BCL6* | *BCOR* | *BCORL1* | *BLM* | *BRAF* | *BRCA1* | *BRCA2* | *BRD4* |
| *BRIP1* | *BTG1* | *BTK* | *C11orf30* | *CARD11* | *CBFB* | *CBL* | *CCND1* | *CCND2* |
| *CCND3* | *CCNE1* | *CD274* | *CD79A* | *CD79B* | *CDC73* | *CDH1* | *CDK12* | *CDK4* |
| *CDK6* | *CDK8* | *CDKN1A* | *CDKN1B* | *CDKN2A* | *CDKN2B* | *CDKN2C* | *CEBPA* | *CHD2* |
| *CHD4* | *CHEK1* | *CHEK2* | *CIC* | *CREBBP* | *CRKL* | *CRLF2* | *CSF1R* | *CTCF* |
| *CTNNA1* | *CTNNB1* | *CUL3* | *CYLD* | *DAXX* | *DDR2* | *DICER1* | *DNMT3A* | *DOT1L* |
| *EGFR* | *EP300* | *EPHA3* | *EPHA5* | *EPHA7* | *EPHB1* | *ERBB2* | *ERBB3* | *ERBB4* |
| *ERG* | *ERRFI1* | *ESR1* | *EZH2* | *FAM46C* | *FANCA* | *FANCC* | *FANCD2* | *FANCE* |
| *FANCF* | *FANCG* | *FANCL* | *FAS* | *FAT1* | *FBXW7* | *FGF10* | *FGF14* | *FGF19* |
| *FGF23* | *FGF3* | *FGF4* | *FGF6* | *FGFR1* | *FGFR2* | *FGFR3* | *FGFR4* | *FH* |
| *FLCN* | *FLT1* | *FLT3* | *FLT4* | *FOXL2* | *FOXP1* | *FRS2* | *FUBP1* | *GABRA6* |
| *GATA1* | *GATA2* | *GATA3* | *GATA4* | *GATA6* | *GID4* | *GLI1* | *GNA11* | *GNA13* |
| *GNAQ* | *GNAS* | *GPR124* | *GRIN2A* | *GRM3* | *GSK3B* | *H3F3A* | *HGF* | *HNF1A* |
| *HRAS* | *HSD3B1* | *HSP90AA1* | *IDH1* | *IDH2* | *IGF1R* | *IGF2* | *IKBKE* | *IKZF1* |
| *IL7R* | *INHBA* | *INPP4B* | *IRF2* | *IRF4* | *IRS2* | *JAK1* | *JAK2* | *JAK3* |
| *JUN* | *KAT6A* | *KDM5A* | *KDM5C* | *KDM6A* | *KDR* | *KEAP1* | *KEL* | *KIT* |
| *KLHL6* | *KMT2A* | *KMT2C* | *KMT2D* | *KRAS* | *LMO1* | *LRP1B* | *LYN* | *LZTR1* |
| *MAGI2* | *MAP2K1* | *MAP2K2* | *MAP2K4* | *MAP3K1* | *MCL1* | *MDM2* | *MDM4* | *MED12* |
| *MEF2B* | *MEN1* | *MET* | *MITF* | *MLH1* | *MPL* | *MRE11A* | *MSH2* | *MSH6* |
| *MTOR* | *MUTYH* | *MYC* | *MYCL* | *MYCN* | *MYD88* | *NF1* | *NF2* | *NFE2L2* |
| *NFKBIA* | *NKX2-1* | *NOTCH1* | *NOTCH2* | *NOTCH3* | *NPM1* | *NRAS* | *NSD1* | *NTRK1* |
| *NTRK2* | *NTRK3* | *NUP93* | *PAK3* | *PALB2* | *PARK2* | *PAX5* | *PBRM1* | *PDCD1LG2* |
| *PDGFRA* | *PDGFRB* | *PDK1* | *PIK3C2B* | *PIK3CA* | *PIK3CB* | *PIK3CG* | *PIK3R1* | *PIK3R2* |
| *PLCG2* | *PMS2* | *POLD1* | *POLE* | *PPP2R1A* | *PRDM1* | *PREX2* | *PRKAR1A* | *PRKCI* |
| *PRKDC* | *PRSS8* | *PTCH1* | *PTEN* | *PTPN11* | *QKI* | *RAC1* | *RAD50* | *RAD51* |
| *RAF1* | *RANBP2* | *RARA* | *RB1* | *RBM10* | *RET* | *RICTOR* | *RNF43* | *ROS1* |
| *RPTOR* | *RUNX1* | *RUNX1T1* | *SDHA* | *SDHB* | *SDHC* | *SDHD* | *SETD2* | *SF3B1* |
| *SLIT2* | *SMAD2* | *SMAD3* | *SMAD4* | *SMARCA4* | *SMARCB1* | *SMO* | *SNCAIP* | *SOCS1* |
| *SOX10* | *SOX2* | *SOX9* | *SPEN* | *SPOP* | *SPTA1* | *SRC* | *STAG2* | *STAT3* |
| *STAT4* | *STK11* | *SUFU* | *SYK* | *TAF1* | *TBX3* | *TERC* | *TERT** | *TET2* |
| *TGFBR2* | *TNFAIP3* | *TNFRSF14* | *TOP1* | *TOP2A* | *TP53* | *TSC1* | *TSC2* | *TSHR* |
| *U2AF1* | *VEGFA* | *VHL* | *WISP3* | *WT1* | *XPO1* | *ZBTB2* | *ZNF217* | *ZNF703* |
| **REARRANGEMENTS** | | | | | | | | |
| *ALK* | *BCL2* | *BCR* | *BRAF* | *BRCA1* | *BRCA2* | *BRD4* | *EGFR* | *ETV1* |
| *ETV4* | *ETV5* | *ETV6* | *FGFR1* | *FGFR2* | *FGFR3* | *KIT* | *MSH2* | *MYB* |
| *MYC* | *NOTCH2* | *NTRK1* | *NTRK2* | *PDGFRA* | *RAF1* | *RARA* | *RET* | *ROS1* |
| *TMPRSS2* |  |  |  |  |  |  |  |  |

**TERT* promoter region

**Panel S2C**. 324 gene panel of tissue-DNA (*Foundation Medicine, Inc*) (N=5 tissue samples).

| **SUBSTITUTIONS, INSERTION/DELETIONS, AND COPY NUMBER ALTERATIONS** | | | | | | | | |
| --- | --- | --- | --- | --- | --- | --- | --- | --- |
| *ABL1* | *ACVR1B* | *AKT1* | *AKT2* | *AKT3* | *ALK* | *ALOX12B* | *AMER1* | *APC* |
| *AR* | *ARAF* | *ARFRP1* | *ARID1A* | *ASXL1* | *ATM* | *ATR* | *ATRX* | *AURKA* |
| *AURKB* | *AXIN1* | *AXL* | *BAP1* | *BARD1* | *BCL2* | *BCL2L1* | *BCL2L2* | *BCL6* |
| *BCOR* | *BCORL1* | *BRAF* | *BRCA1* | *BRCA2* | *BRD4* | *BRIP1* | *BTG1* | *BTG2* |
| *BTK* | *C11orf30* | *C17orf39* | *CALR* | *CARD11* | *CASP8* | *CBFB* | *CBL* | *CCND1* |
| *CCND2* | *CCND3* | *CCNE1* | *CD22* | *CD274* | *CD70* | *CD79A* | *CD79B* | *CDC73* |
| *CDH1* | *CDK12* | *CDK4* | *CDK6* | *CDK8* | *CDKN1A* | *CDKN1B* | *CDKN2A* | *CDKN2B* |
| *CDKN2C* | *CEBPA* | *CHEK1* | *CHEK2* | *CIC* | *CREBBP* | *CRKL* | *CSF1R* | *CSF3R* |
| *CTCF* | *CTNNA1* | *CTNNB1* | *CUL3* | *CUL4A* | *CXCR4* | *CYP17A1* | *DAXX* | *DDR1* |
| *DDR2* | *DIS3* | *DNMT3A* | *DOT1L* | *EED* | *EGFR* | *EP300* | *EPHA3* | *EPHB1* |
| *EPHB4* | *ERBB2* | *ERBB3* | *ERBB4* | *ERCC4* | *ERG* | *ERRFI1* | *ESR1* | *EZH2* |
| *FAM46C* | *FANCA* | *FANCC* | *FANCG* | *FANCL* | *FAS* | *FBXW7* | *FGF10* | *FGF12* |
| *FGF14* | *FGF19* | *FGF23* | *FGF3* | *FGF4* | *FGF6* | *FGFR1* | *FGFR2* | *FGFR3* |
| *FGFR4* | *FH* | *FLCN* | *FLT1* | *FLT3* | *FOXL2* | *FUBP1* | *GABRA6* | *GATA3* |
| *GATA4* | *GATA6* | *GNA11* | *GNA13* | *GNAQ* | *GNAS* | *GRM3* | *GSK3B* | *H3F3A* |
| *HDAC1* | *HGF* | *HNF1A* | *HRAS* | *HSD3B1* | *ID3* | *IDH1* | *IDH2* | *IGF1R* |
| *IKBKE* | *IKZF1* | *INPP4B* | *IRF2* | *IRF4* | *IRS2* | *JAK1* | *JAK2* | *JAK3* |
| *JUN* | *KDM5A* | *KDM5C* | *KDM6A* | *KDR* | *KEAP1* | *KEL* | *KIT* | *KLHL6* |
| *KMT2A* | *KMT2D* | *KRAS* | *LTK* | *LYN* | *MAF* | *MAP2K1* | *MAP2K2* | *MAP2K4* |
| *MAP3K1* | *MAP3K13* | *MAPK1* | *MCL1* | *MDM2* | *MDM4* | *MED12* | *MEF2B* | *MEN1* |
| *MERTK* | *MET* | *MITF* | *MKNK1* | *MLH1* | *MPL* | *MRE11A* | *MSH2* | *MSH3* |
| *MSH6* | *MST1R* | *MTAP* | *MTOR* | *MUTYH* | *MYC* | *MYCL* | *MYCN* | *MYD88* |
| *NBN* | *NF1* | *NF2* | *NFE2L2* | *NFKBIA* | *NKX2-1* | *NOTCH1* | *NOTCH2* | *NOTCH3* |
| *NPM1* | *NRAS* | *NSD3* | *NT5C2* | *NTRK1* | *NTRK2* | *NTRK3* | *P2RY8* | *PALB2* |
| *PARK2* | *PARP1* | *PARP2* | *PARP3* | *PAX5* | *PBRM1* | *PDCD1* | *PDCD1LG2* | *PDGFRA* |
| *PDGFRB* | *PDK1* | *PIK3C2B* | *PIK3C2G* | *PIK3CA* | *PIK3CB* | *PIK3R1* | *PIM1* | *PMS2* |
| *POLD1* | *POLE* | *PPARG* | *PPP2R1A* | *PPP2R2A* | *PRDM1* | *PRKAR1A* | *PRKCI* | *PTCH1* |
| *PTEN* | *PTPN11* | *PTPRO* | *QKI* | *RAC1* | *RAD21* | *RAD51* | *RAD51B* | *RAD51C* |
| *RAD51D* | *RAD52* | *RAD54L* | *RAF1* | *RARA* | *RB1* | *RBM10* | *REL* | *RET* |
| *RICTOR* | *RNF43* | *ROS1* | *RPTOR* | *SDHA* | *SDHB* | *SDHC* | *SDHD* | *SETD2* |
| *SF3B1* | *SGK1* | *SMAD2* | *SMAD4* | *SMARCA4* | *SMARCB1* | *SMO* | *SNCAIP* | *SOCS1* |
| *SOX2* | *SOX9* | *SPEN* | *SPOP* | *SRC* | *STAG2* | *STAT3* | *STK11* | *SUFU* |
| *SYK* | *TBX3* | *TEK* | *TET2* | *TGFBR2* | *TIPARP* | *TNFAIP3* | *TNFRSF14* | *TP53* |
| *TSC1* | *TSC2* | *TYRO3* | *U2AF1* | *VEGFA* | *VHL* | *WHSC1* | *WT1* | *XPO1* |
| *XRCC2* | *ZNF217* | *ZNF703* |  |  |  |  |  |  |
| **REARRANGEMENTS** | | | | | | | | |
| *ALK* | *BCL2* | *BCR* | *BRAF* | *BRCA1* | *BRCA2* | *CD74* | *EGFR* | *ETV4* |
| *ETV5* | *ETV6* | *EWSR1* | *EZR* | *FGFR1* | *FGFR2* | *FGFR3* | *KIT* | *KMT2A* |
| *MSH2* | *MYB* | *MYC* | *NOTCH2* | *NTRK1* | *NTRK2* | *NUTM1* | *PDGFRA* | *RAF1* |
| *RARA* | *RET* | *ROS1* | *RSPO2* | *SDC4* | *SLC34A2* | *TERC* | *TERT** | *TMPRSS2* |

**TERT* promoter region

**Panel S2D**. 397 gene panel of tissue-DNA (UC San Diego Health Clinical Laboratories) (N=5 tissue samples).

| **SINGLE NUCLEOTIDE, SMALL INSERTIONS, OR DELETIONS** | | | | | | | | | |
| --- | --- | --- | --- | --- | --- | --- | --- | --- | --- |
| *ABL1* | *ABL2* | *ACVR1B* | *AKAP9* | *AKT1* | *AKT2* | *AKT3* | *ALK* | *AMER1* | *APC* |
| *AR* | *ARAF* | *ARFRP1* | *ARID1A* | *ARID1B* | *ARID2* | *ASPSCR1* | *ASXL1* | *ATF6* | *ATM* |
| *ATP1A1* | *ATP2B3* | *ATR* | *ATRX* | *AURKA* | *AURKB* | *AXIN1* | *AXL* | *BAP1* | *BARD1* |
| *BCL11A* | *BCL11B* | *BCL2* | *BCL2L1* | *BCL2L2* | *BCL6* | *BCOR* | *BCORL1* | *BLM* | *BMPR1A* |
| *BRAF* | *BRCA1* | *BRCA2* | *BRD4* | *BRIP1* | *BTG1* | *BTK* | *C11orf30* | *CACNA1D* | *CAMTA1* |
| *CARD11* | *CASP8* | *CBFB* | *CBL* | *CBLB* | *CCND1* | *CCND2* | *CCND3* | *CCNE1* | *CD274* |
| *CD79A* | *CD79B* | *CDC73* | *CDH1* | *CDH11* | *CDK12* | *CDK4* | *CDK6* | *CDK8* | *CDKN1A* |
| *CDKN1B* | *CDKN2A* | *CDKN2B* | *CDKN2C* | *CEBPA* | *CHD2* | *CHD4* | *CHEK1* | *CHEK2* | *CIC* |
| *CLTCL1* | *COL1A1* | *CREBBP* | *CRKL* | *CRLF2* | *CSF1R* | *CSF3R* | *CTCF* | *CTNNA1* | *CTNNB1* |
| *CUL3* | *CYLD* | *DAXX* | *DDIT3* | *DDR2* | *DICER1* | *DNM2* | *DNMT3A* | *DOT1L* | *EBF1* |
| *EGFR* | *EIF1AX* | *EP300* | *EPHA3* | *EPHA5* | *EPHA7* | *EPHB1* | *ERBB2* | *ERBB3* | *ERBB4* |
| *ERCC3* | *ERCC4* | *ERCC5* | *ERG* | *ERRFI1* | *ESR1* | *ETV1* | *ETV4* | *ETV5* | *ETV6* |
| *EWSR1* | *EXT1* | *EZH2* | *FAM46C* | *FANCA* | *FANCC* | *FANCD2* | *FANCE* | *FANCF* | *FANCG* |
| *FANCL* | *FAS* | *FAT1* | *FBXO11* | *FBXW7* | *FGF10* | *FGF14* | *FGF19* | *FGF23* | *FGF3* |
| *FGF4* | *FGF6* | *FGFR1* | *FGFR2* | *FGFR3* | *FGFR4* | *FH* | *FLCN* | *FLT1* | *FLT3* |
| *FLT4* | *FOXA1* | *FOXL2* | *FOXO1* | *FOXP1* | *FRS2* | *FUBP1* | *FUS* | *GABRA6* | *GATA1* |
| *GATA2* | *GATA3* | *GATA4* | *GATA6* | *GID4* | *GLI1* | *GMPS* | *GNA11* | *GNA13* | *GNAQ* |
| *GNAS* | *GPR124* | *GRIN2A* | *GRM3* | *GSK3B* | *H3F3A* | *HGF* | *HIP1* | *HNF1A* | *HRAS* |
| *HSD3B1* | *HSP90AA1* | *IDH1* | *IDH2* | *IGF1R* | *IGF2* | *IKBKE* | *IKZF1* | *IL7R* | *INHBA* |
| *INPP4B* | *IRF2* | *IRF4* | *IRS2* | *ITK* | *JAK1* | *JAK2* | *JAK3* | *JUN* | *KAT6A* |
| *KAT6B* | *KDM5A* | *KDM5C* | *KDM6A* | *KDR* | *KEAP1* | *KEL* | *KIF5B* | *KIT* | *KLHL6* |
| *KMT2A* | *KMT2D* | *KRAS* | *LCP1* | *LIFR* | *LMO1* | *LRIG3* | *LRP1B* | *LYN* | *LZTR1* |
| *MAGI2* | *MAML2* | *MAP2K1* | *MAP2K2* | *MAP2K4* | *MAP3K1* | *MCL1* | *MDM2* | *MDM4* | *MED12* |
| *MEF2B* | *MEN1* | *MET* | *MITF* | *MLH1* | *MLLT3* | *MLLT4* | *MN1* | *MPL* | *MRE11A* |
| *MSH2* | *MSH6* | *MTOR* | *MUTYH* | *MYB* | *MYC* | *MYCL* | *MYCN* | *MYD88* | *MYH11* |
| *MYH9* | *NCOA1* | *NCOA2* | *NF1* | *NF2* | *NFE2L2* | *NFKBIA* | *NIN* | *NKX2-1* | *NOTCH1* |
| *NOTCH2* | *NOTCH3* | *NPM1* | *NR4A3* | *NRAS* | *NSD1* | *NTRK1* | *NTRK2* | *NTRK3* | *NUMA1* |
| *NUP214* | *NUP93* | *NUP98* | *PAK3* | *PALB2* | *PARK2* | *PAX3* | *PAX5* | *PAX7* | *PBRM1* |
| *PCM1* | *PDCD1LG2* | *PDGFRA* | *PDGFRB* | *PDK1* | *PIK3C2B* | *PIK3CA* | *PIK3CB* | *PIK3CG* | *PIK3R1* |
| *PIK3R2* | *PLCG2* | *PMS1* | *PMS2* | *POLD1* | *POLE* | *PPARG* | *PPP2R1A* | *PRDM1* | *PRDM16* |
| *PREX2* | *PRKAR1A* | *PRKCI* | *PRKDC* | *PRSS8* | *PTCH1* | *PTEN* | *PTPN11* | *PTPRC* | *QKI* |
| *RAC1* | *RAD21* | *RAD50* | *RAD51* | *RAF1* | *RALGDS* | *RANBP17* | *RANBP2* | *RARA* | *RB1* |
| *RBM10* | *RET* | *RICTOR* | *RNF43* | *ROS1* | *RPTOR* | *RUNX1* | *RUNX1T1* | *SDHA* | *SDHB* |
| *SDHC* | *SDHD* | *SETBP1* | *SETD2* | *SF3B1* | *SLC34A2* | *SLIT2* | *SMAD2* | *SMAD3* | *SMAD4* |
| *SMARCA4* | *SMARCB1* | *SMO* | *SNCAIP* | *SOCS1* | *SOX10* | *SOX2* | *SOX9* | *SPEN* | *SPOP* |
| *SPTA1* | *SRC* | *SRGAP3* | *SS18* | *STAG2* | *STAT3* | *STAT4* | *STAT5B* | *STK11* | *SUFU* |
| *SYK* | *TAF1* | *TBX3* | *TCF7L2* | *TERC* | *TERT* | *TET2* | *TGFBR2* | *THRAP3* | *TMPRSS2* |
| *TNFAIP3* | *TNFRSF14* | *TOP1* | *TOP2A* | *TP53* | *TPR* | *TRIM24* | *TRIM33* | *TRIP11* | *TRRAP* |
| *TSC1* | *TSC2* | *TSHR* | *U2AF1* | *VEGFA* | *VHL* | *WHSC1* | *WISP3* | *WRN* | *WT1* |
| *XPO1* | *ZBTB2* | *ZMYM2* | *ZNF217* | *ZNF384* | *ZNF521* | *ZNF703* |  |  |  |
| **REARRANGEMENTS** | | | | | | | | | |
| *ALK* | *ASPSCR1* | *BRAF* | *BRD4* | *DDIT3* | *EGFR* | *ETV1* | *ETV4* | *ETV5* | *ETV6* |
| *EWSR1* | *FGFR1* | *FGFR2* | *FGFR3* | *FOXO1* | *FUS* | *MYB* | *NOTCH2* | *NR4A3* | *NTRK1* |
| *NTRK2* | *PDGFRA* | *PPARG* | *RAF1* | *RET* | *ROS1* | *SS18* | *TMPRSS2* |  |  |

**Supplementary Table S3.** Detailed information of invasive glioma patients harboring characterized cfDNA alterations (N=28).

| **ID** | **Diagnosis**  **(WHO grade)** | **Age*** | **Characterized alterations in cfDNA NGS (%cfDNA)** | **Prior systemic treatment to blood draw for cfDNA** | **Characterized alterations**  **in tissue DNA NGS** |
| --- | --- | --- | --- | --- | --- |
| ***Both cfDNA and Tissue DNA available (N=18)*** | | | | | |
| 1 | GBM (IV) | 60 | *ATM* R3008H (0.2%) | TMZ+RT | *FGFR3-TACC3* fusion, *TERT* promoter -124C>T |
| 6 | GBM (IV) | 70 | *TP53* D281E (4.2%) | TMZ+RT; antineoplaston therapy | *EGFR* amplification, *PTEN* R159fs*21, *CDKN2A*/B loss, *TERT* promoter -124C>T |
| 14 | AA (III) | 35 | *TP53* Y163C (0.1%) | TMZ+RT; lomustine; bevacizumab+irinotecan | *TP53* P278S, *TP53* R249T, *IDH1* R132S, *ATRX* N1232fs*15 |
| 23 | AA (III) | 52 | *TP53* V143M (0.4%) | TMZ+RT | *TP53* P142fs*28, *TP53* S241A, *IDH1* R132H, *ATRX* K1305fs*41 |
| 27 | GBM (IV) | 71 | *TP53* Y234C (0.7%) | TMZ+RT | *CSF1R* V32G, *PTEN* I101T |
| 44 | GBM (IV) | 62 | *ATM* R3008H (0.1%) | TMZ+RT | *TERT* promoter -124C>T, *PTEN* loss, *CDKN2C* loss, *CDKN2A*/B loss |
| 50 | AO (III) | 45 | *PDGFRA* R500* (0.62%) | TMZ+RT; lomustine; carboplatin | *NF1* Y2285fs*5, *NF1* F1275fs*8, *NF1* Y1296*, *PIK3CA* Q546R, *PIK3R1* splice site 1743-2A>G, *IDH1* R132H |
| 54 | GBM (IV) | 70 | *TP53* Y220C (3.0%), *TP53* P152L (0.31%) | TMZ+RT | *NF1* loss, *PTEN* loss exons 2-9, *TSC2* loss, *ATR* R1082H, *TP53* R158H, *RB1* loss, *TERT* promoter -124C>T |
| 59 | GBM (IV) | 70 | *TP53* L265P (0.81%) | TMZ+RT; mipsagargin; nilotinib; bevacizumab+lomustine | *BRAF* D594N, *EGFR* A289V, *EGFR* amplification, EGFRvIII, *PIK3CA* G1049R, *CDKN2A*/B loss, |
| 95 | GBM (IV) | 41 | *TP53* S215R (2.34%) | TMZ+RT; bevacizumab+carboplatin; bevacizumab+lomustine | *CDK4* amplification, *PTEN* R130*, *MDM2* amplification, *ARID2* S660*, *TERT* promoter -124C>T |
| 101 | GBM (IV) | 61 | *TP53* V143M (0.2%) | TMZ+RT | *NF1* K938*, *PTEN* N276fs*15 |
| 105 | AA (III) | 25 | *IDH1* R132H (2%) | TMZ+RT | *IDH1* R132H, *NOTCH1* F357del, *TP53* E285K, *TP53* splice site 719_782+43del107, *ATRX* D2010fs*1, *FUBP1* Q365* |
| 107 | AA (III) | 81 | *JAK2* V617F (0.3%) | TMZ+RT | *EGFR* amplification, EGFRvIII, *CDKN2A*/B loss, *TERT* promoter-124C>T |
| 111 | GBM (IV) | 48 | *TP53* R249S (0.3%) | TMZ+RT | *EGFR* A289V, *EGFR* amplification, *EGFR* V292L, *PTEN* R130*, *CDKN2A*/B loss, *MTAP* loss exons 2-8, *STAG2* R259*, *TERT* promoter -124C>T |
| 114 | OD (II) | 43 | *GNAS* R201C (0.4%) | No systemic treatment (surgical resection and radiation) | *IDH1* R132H, *ERBB4* R838Q, *TERT* promoter -146C>T |
| 120 | GBM (IV) | 54 | *EGFR* A289V (0.5%), *TP53* S241F (0.2%), *TP53* I254S (0.1%) | TMZ+RT; bevacizumab | *EGFR* A289V, *EGFR* amplification, EGFRvIII, *MDM4* amplification, *CDKN2A*/B loss, *PIK3C2B* amplification |
| 123 | GBM (IV) | 59 | *JAK2* V617F (0.3%), *GNAS* R201H (0.3%) | TMZ+RT; optune+TMZ | *PTEN* Splice site axon 9, *RB1* E675Sfs*16, *TP53* R249S |
| 125 | AA (III) | 40 | *TP53* Y220C (0.9%), *TP53* C238_M243del (0.1%) | TMZ+RT; lomustine+procarbazine+vincristine; bevacizumab+irinotecan | *NF1* F1247fs*18, *NF1* I275fs*14, *PTCH1* G1212S, *IDH1* R132S, *ATRX* T1582fs*24, *TP53* R249G |
| ***Only cfDNA available (N=10)*** | | | | | |
| 2 | GBM (IV) | 61 | *MET* amplification** | TMZ+RT; bevacizumab+carboplatin | No tissue NGS results |
| 16 | GBM (IV) | 45 | *JAK2* V617F (0.3%) | TMZ+RT | No tissue NGS results |
| 30 | GBM (IV) | 56 | *TP53* P152S (1.2%) | TMZ+RT | No tissue NGS results |
| 38 | GBM (IV) | 67 | *BRAF* Q257R (0.5%) | TMZ+RT | No tissue NGS results |
| 66 | GBM (IV) | 47 | *BRCA1* Q380* (0.11%) | TMZ+RT; bevacizumab | No tissue NGS results |
| 79 | GBM (IV) | 66 | *TP53* R280I (0.2%) | TMZ+RT; mipsagargin; nilotinib | No tissue NGS results |
| 82 | GBM (IV) | 54 | *TP53* C238S (1.41%) | TMZ+RT; bevacizumab+lomustine | No tissue NGS results |
| 113 | GBM (IV) | 63 | *TP53* E258K (0.2%), *FBXW7* R465H (0.1%) | TMZ+RT; bevacizumab; bevacizumab+lomustine | No tissue NGS results |
| 115 | GBM (IV) | 59 | *TP53* G245S (2.9%), *TP53* R213* (0.3%) | TMZ+RT | No tissue NGS results |
| 128 | GBM (IV) | 59 | *TP53* F134C (0.4%), *TP53* G199E (0.2%), *NF1* G672fs (0.1%) | TMZ+RT | No tissue NGS results |

* At the time of blood draw for cfDNA (years).

** %cfDNA is not calculated for genomic amplifications.

**Abbreviations**: AA, anaplastic astrocytoma; AO, anaplastic oligodendroglioma; cfDNA, cell-free DNA; GBM, glioblastoma multiforme; OD, oligodendroglioma; TMZ+RT, temozolomide with concurrent radiation therapy; %cfDNA, mutant allele frequency.

**Supplementary Table S4.** Presence of characterized cfDNA alterations and overall survival time from blood draw for cfDNA in invasive glioma patients (N=135).

| **ID** | **Diagnosis (WHO grade)** | **Age*** | **Characterized cfDNA alterations detected** | **OS time from blood draw for cfDNA** |  | **ID** | **Diagnosis (WHO grade)** | **Age*** | **Characterized cfDNA alterations detected** | **OS time from blood draw for cfDNA** |
| --- | --- | --- | --- | --- | --- | --- | --- | --- | --- | --- |
| 1 | GBM (IV) | 60 | YES | 10.8 |  | 41 | GBM (IV) | 65 | NO | 18.8 |
| 2 | GBM (IV) | 61 | YES | 11.9 |  | 42 | AA (III) | 31 | NO | 21.7+ |
| 3 | GBM (IV) | 45 | NO | 17.3 |  | 43 | GBM (IV) | 77 | NO | 23.4 |
| 4 | GBM (IV) | 57 | NO | 29.9 |  | 44 | GBM (IV) | 62 | YES | 2.3 |
| 5 | GBM (IV) | 53 | NO | 9.8 |  | 45 | GBM (IV) | 37 | NO | 6.5 |
| 6 | GBM (IV) | 70 | YES | 34.8 |  | 46 | GBM (IV) | 71 | NO | 46.9+ |
| 7 | GBM (IV) | 81 | NO | 1.7+ |  | 47 | GBM (IV) | 63 | NO | 15.1 |
| 8 | GBM (IV) | 75 | NO | 2.7 |  | 48 | GBM (IV) | 65 | NO | 44.4 |
| 9 | OD (II) | 37 | NO | 16.1+ |  | 49 | GBM (IV) | 53 | NO | 4.2 |
| 10 | GBM (IV) | 87 | NO | 8.7+ |  | 50 | AO (III) | 45 | YES | 20+ |
| 11 | GBM (IV) | 46 | NO | 12.5 |  | 51 | GBM (IV) | 70 | NO | 17.7 |
| 12 | GBM (IV) | 31 | NO | 41.7 |  | 52 | OD (II) | 52 | NO | 14.3+ |
| 13 | GBM (IV) | 46 | NO | 6.3 |  | 53 | GBM (IV) | 48 | NO | 2.2 |
| 14 | AA (III) | 35 | YES | 6.1 |  | 54 | GBM (IV) | 70 | YES | 11.8 |
| 15 | OD (II) | 69 | NO | 2.1+ |  | 55 | GBM (IV) | 57 | NO | 5.2 |
| 16 | GBM (IV) | 45 | YES | 7.6 |  | 56 | DA (II) | 53 | NO | 16.3 |
| 17 | GBM (IV) | 63 | NO | 9.6 |  | 57 | AO (III) | 53 | NO | 0.1+ |
| 18 | GBM (IV) | 78 | NO | 4.9 |  | 58 | GBM (IV) | 69 | NO | 24.4 |
| 19 | GBM (IV) | 37 | NO | 28 |  | 59 | GBM (IV) | 70 | YES | 2.6 |
| 20 | GBM (IV) | 58 | NO | 1.8 |  | 60 | AA (III) | 67 | NO | 49.5+ |
| 21 | AA (III) | 40 | NO | 51.6+ |  | 61 | AA (III) | 29 | NO | 24.2 |
| 22 | AA (III) | 51 | NO | 16.6+ |  | 62 | GBM (IV) | 64 | NO | 18.5 |
| 23 | AA (III) | 52 | YES | 52+ |  | 63 | GBM (IV) | 44 | NO | 15.9 |
| 24 | GBM (IV) | 24 | NO | 6.5 |  | 64 | OD (II) | 61 | NO | 49+ |
| 25 | AA (III) | 41 | NO | 52+ |  | 65 | GBM (IV) | 82 | NO | 9.9 |
| 26 | GBM (IV) | 30 | NO | 7.1 |  | 66 | GBM (IV) | 47 | YES | 16.5 |
| 27 | GBM (IV) | 71 | YES | 50.3+ |  | 67 | OD (II) | 66 | NO | 2.5 |
| 28 | GBM (IV) | 49 | NO | 10.5 |  | 68 | GBM (IV) | 48 | NO | 47.3+ |
| 29 | GBM (IV) | 52 | NO | 11.3 |  | 69 | GBM (IV) | 71 | NO | 2.7 |
| 30 | GBM (IV) | 56 | YES | 3.5 |  | 70 | GBM (IV) | 76 | NO | 35.7 |
| 31 | AA (III) | 43 | NO | 50+ |  | 71 | DA (II) | 29 | NO | 47.1+ |
| 32 | GBM (IV) | 46 | NO | 12.5 |  | 72 | DA (II) | 31 | NO | 34.7 |
| 33 | AO (III) | 62 | NO | 4.3 |  | 73 | GBM (IV) | 73 | NO | 27.2+ |
| 34 | GBM (IV) | 66 | NO | 9.2+ |  | 74 | AA (III) | 50 | NO | 15.7 |
| 35 | GBM (IV) | 64 | NO | 5.8 |  | 75 | AA (III) | 54 | NO | 47.2+ |
| 36 | GBM (IV) | 38 | NO | 18.5 |  | 76 | AA (III) | 34 | NO | 49.8+ |
| 37 | GBM (IV) | 56 | NO | 32.9 |  | 77 | GBM (IV) | 26 | NO | 16.1 |
| 38 | GBM (IV) | 67 | YES | 2.7 |  | 78 | OD (II) | 61 | NO | 16.3 |
| 39 | AO (III) | 42 | NO | 5.5 |  | 79 | GBM (IV) | 66 | YES | 18.1 |
| 40 | OD (II) | 56 | NO | 49.9+ |  | 80 | AA (III) | 63 | NO | 49.7+ |

(Continued on following page)

| **ID** | **Diagnosis (WHO grade)** | **Age*** | **Characterized cfDNA alterations detected** | **OS time form blood draw for cfDNA** |  | **ID** | **Diagnosis (WHO grade)** | **Age*** | **Characterized cfDNA alterations detected** | **OS time form blood draw for cfDNA** |
| --- | --- | --- | --- | --- | --- | --- | --- | --- | --- | --- |
| 81 | DA (II) | 65 | NO | 29.2 |  | 121 | GBM (IV) | 44 | NO | 3 |
| 82 | GBM (IV) | 54 | YES | 3.8 |  | 122 | GBM (IV) | 51 | NO | 18.1 |
| 83 | DA (II) | 63 | NO | 17.7 |  | 123 | GBM (IV) | 59 | YES | 3.8 |
| 84 | GBM (IV) | 35 | NO | 7.3 |  | 124 | AA (III) | 55 | NO | 3.8 |
| 85 | DA (II) | 38 | NO | 19.7 |  | 125 | AA (III) | 40 | YES | 6.4 |
| 86 | GBM (IV) | 62 | NO | 4 |  | 126 | GBM (IV) | 62 | NO | 8.2 |
| 87 | GBM (IV) | 60 | NO | 5.3 |  | 127 | DA (II) | 55 | NO | 9 |
| 88 | GBM (IV) | 78 | NO | 1.9+ |  | 128 | GBM (IV) | 59 | YES | 11.5 |
| 89 | GBM (IV) | 69 | NO | 27.1 |  | 129 | GBM (IV) | 60 | NO | 11.2+ |
| 90 | GBM (IV) | 62 | NO | 16.7 |  | 130 | GBM (IV) | 59 | NO | 13.4 |
| 91 | OD (II) | 32 | NO | 50.2+ |  | 131 | GBM (IV) | 71 | NO | 1.8 |
| 92 | GBM (IV) | 58 | NO | 11.9 |  | 132 | GBM (IV) | 58 | NO | 9.3 |
| 93 | AA (III) | 23 | NO | 47.4+ |  | 133 | GBM (IV) | 53 | NO | 8+ |
| 94 | GBM (IV) | 60 | NO | 5.9 |  | 134 | GBM (IV) | 66 | NO | 5.2+ |
| 95 | GBM (IV) | 41 | YES | 4.5 |  | 135 | AA (III) | 50 | NO | 2.9+ |
| 96 | GBM (IV) | 45 | NO | 1.1 |  |  |  |  |  |  |
| 97 | GBM (IV) | 53 | NO | 8.9 |  |  |  |  |  |  |
| 98 | OD (II) | 45 | NO | 43+ |  |  |  |  |  |  |
| 99 | GBM (IV) | 66 | NO | 3.7 |  |  |  |  |  |  |
| 100 | GBM (IV) | 49 | NO | 45.1+ |  |  |  |  |  |  |
| 101 | GBM (IV) | 61 | YES | 6.6 |  |  |  |  |  |  |
| 102 | AA (III) | 73 | NO | 9.7+ |  |  |  |  |  |  |
| 103 | AA (III) | 42 | NO | 36.1 |  |  |  |  |  |  |
| 104 | GBM (IV) | 63 | NO | 1.2 |  |  |  |  |  |  |
| 105 | AA (III) | 25 | YES | 35.5+ |  |  |  |  |  |  |
| 106 | GBM (IV) | 49 | NO | 14.2+ |  |  |  |  |  |  |
| 107 | AA (III) | 81 | YES | 9.9 |  |  |  |  |  |  |
| 108 | GBM (IV) | 63 | NO | 16.6 |  |  |  |  |  |  |
| 109 | GBM (IV) | 47 | NO | 7.6+ |  |  |  |  |  |  |
| 110 | GBM (IV) | 80 | NO | 3.9+ |  |  |  |  |  |  |
| 111 | GBM (IV) | 48 | YES | 4.6+ |  |  |  |  |  |  |
| 112 | GBM (IV) | 61 | NO | 6.7 |  |  |  |  |  |  |
| 113 | GBM (IV) | 63 | YES | 0.6 |  |  |  |  |  |  |
| 114 | OD (II) | 43 | YES | 24.3+ |  |  |  |  |  |  |
| 115 | GBM (IV) | 59 | YES | 9.1 |  |  |  |  |  |  |
| 116 | GBM (IV) | 62 | NO | 17.2 |  |  |  |  |  |  |
| 117 | GBM (IV) | 44 | NO | 2.1 |  |  |  |  |  |  |
| 118 | GBM (IV) | 59 | NO | 15.9 |  |  |  |  |  |  |
| 119 | GBM (IV) | 53 | NO | 12.9 |  |  |  |  |  |  |
| 120 | GBM (IV) | 54 | YES | 7.2 |  |  |  |  |  |  |

* At the time of blood draw for cfDNA (years).

**Abbreviations**: AA, anaplastic astrocytoma; AO, anaplastic oligodendroglioma; cfDNA, cell-free DNA; GBM, glioblastoma multiforme; OD, oligodendroglioma; WHO, world health organization.

**Supplementary Figure S1**. Frequency (% of patients) of genomic alterations in tissue-DNA NGS among glioma patients (N=88). Only characterized alterations are shown. A total of 89 different genes were involved (only genes altered in ≥2 samples are shown*).


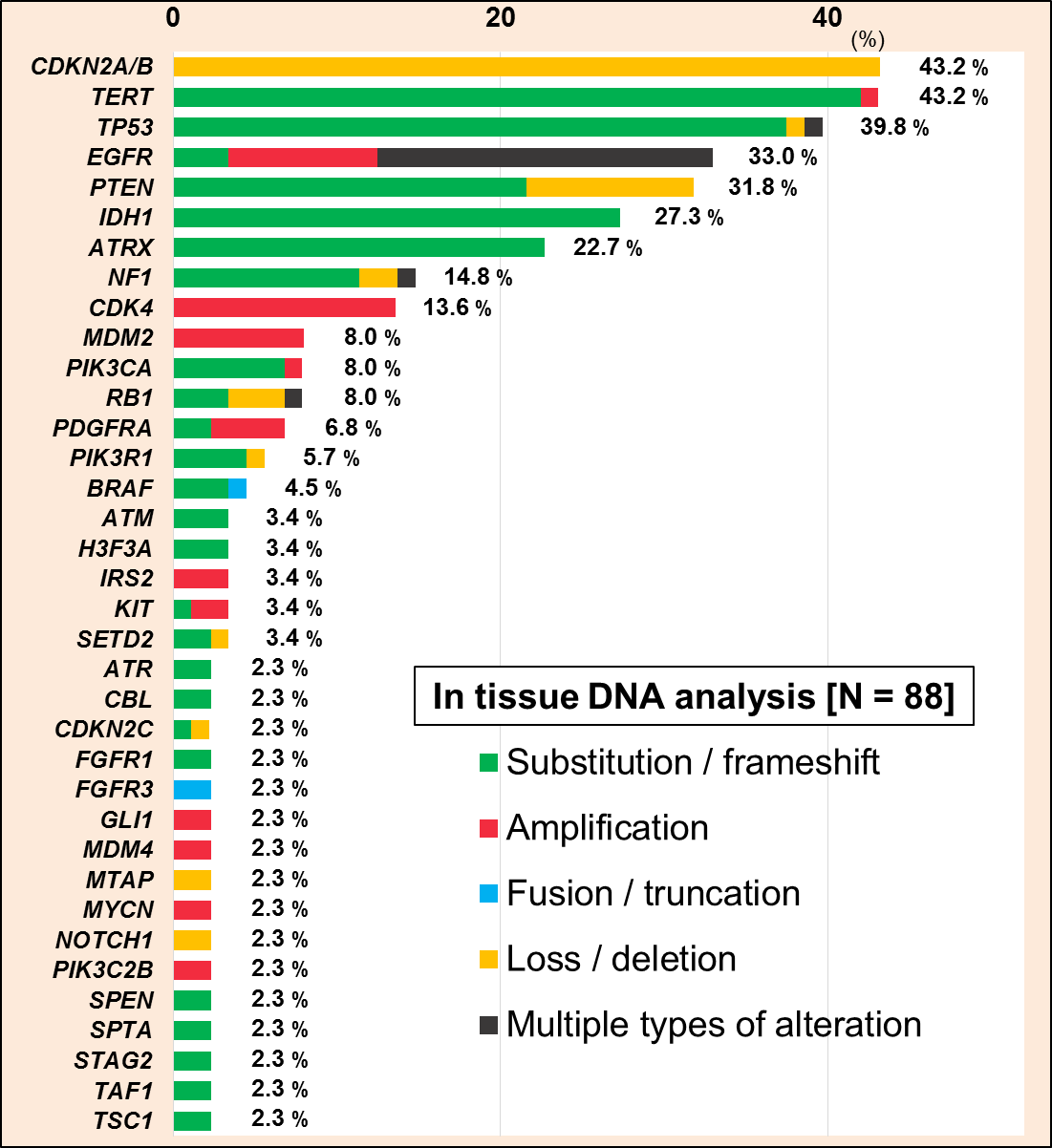


*The following 53 genes were altered in one sample: *ACVR1B, AKT1, AKT3, ARID1A, ARID2, BRIP1, CCNE1, CHD2, CHEK2, CIC, CREBBP, CRKL, CSF1R, CTNNA1, DNMT3A, EP300, ERBB4, FANCA, FANCC, FAS, FAT1, FUBP1, GATA4, HGF, HNF1A, IDH2, JAK2, KDR, KEL, LRP1B, MET, MLL2, MRE11A, MSH2, MUTYH, MYC, NOTCH2, NTRK3, PBRM1, PIK3CG, PLCG2, POLE, PTCH1, PTPN11, QKI, RAD50, SMARCB1, SMO, SOX2, STAT4, TET2, TSC2,* and *WT1.*

**Supplementary Figure S2**. Kaplan-Meier curve for overall survival (OS) from cfDNA blood test depending on the number of characterized cfDNA alterations amongst patients with brain gliomas (N=135).


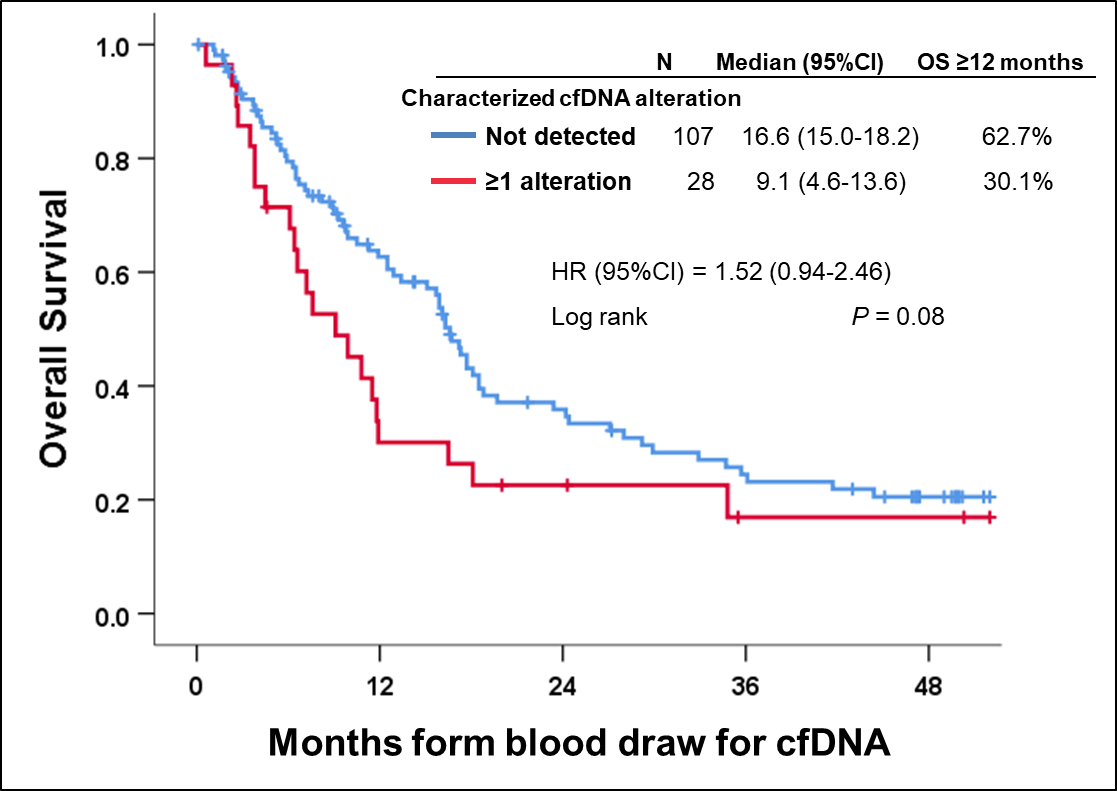


**Supplementary Figure S3**. Detection rate of characterized cfDNA alterations among GBM patients according to presence of tumor diagnosis (N=93).


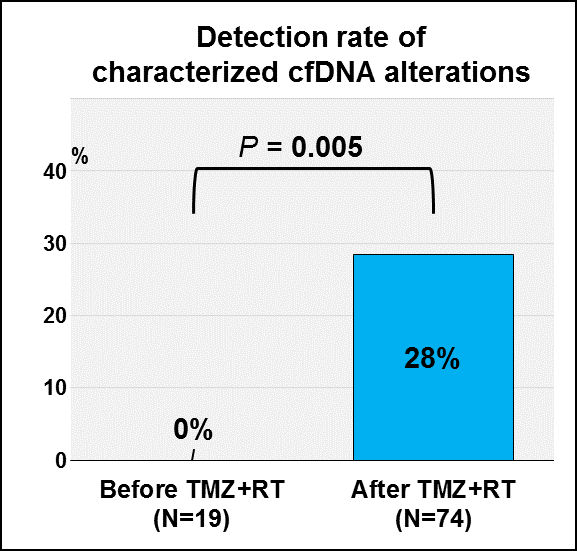

Supplement: Supplementary files [file NIHMS1671969-supplement-Supplementary_files.docx]
