# Supplementary material for: High prevalence of clonal hematopoiesis‐type genomic abnormalities in cell‐free DNA in invasive gliomas after treatment
Source: Int J Cancer. 2021 Feb 5;148(11):2839–47. doi: 10.1002/ijc.33481 (PMC8048515; doi:10.1002/ijc.33481)

SUPPLEMENTARY MATERIALS

High Prevalence of Clonal Hematopoiesis-type Genomic Abnormalities in Cell-free DNA in Invasive Gliomas After Treatment

Ryosuke Okamura[1]*, David E. Piccioni[1, 2]*, Amélie Boichard[1], Suzanna Lee[1], Rebecca E. Jimenez[1], Jason K. Sicklick[1, 3], Shumei Kato[1], and Razelle Kurzrock[1]

CONTENTS

Supplementary Tables

- **Supplementary Table S1.** Next-generation sequencing gene panels for blood-derived cfDNA (*Guardant, Inc*.) (N=135).
- **Supplementary Table S2**. Next-generation sequencing gene panels for tissue-DNA (N=88).
- **Supplementary Table S3.** Detailed information of invasive glioma patients harboring characterized cfDNA alterations (N=28).
- **Supplementary Table S4.** Presence of characterized cfDNA alterations and overall survival time from blood draw for cfDNA in invasive glioma patients (N=135).

Supplementary Figures

- **Supplementary Figure S1**. Frequency (% of patients) of genomic alterations in tissue-DNA NGS among glioma patients (N=88). Only characterized alterations are shown. A total of 89 different genes were involved (only genes altered in ≥2 samples are shown).
- **Supplementary Figure S2**. Kaplan-Meier curve for overall survival (OS) from cfDNA blood test depending on the number of characterized cfDNA alterations amongst patients with brain gliomas (N=135).
- **Supplementary Figure S3**. Detection rate of characterized cfDNA alterations among GBM patients according to presence of tumor diagnosis (N=93).

Supplementary Table S1. Next-generation sequencing gene panels for blood-derived cfDNA (*Guardant, Inc.*) (N=135).

Panel S1A. 54 gene panel (N=45 samples).

**POINT MUTATIONS**				**AMPLIFICATIONS**
ABL1	*AKT1*	***ALK***	***APC***	*EGFR*
AR	*ATM*	***BRAF***	***CDKN2A***	*ERBB2*
CDH1	*CSF1R*	*CTNNB1*	***EGFR***	*MET*
ERBB2	*ERBB4*	*EZH2*	***FBXW7***	
FGFR1	*FGFR2*	*FGFR3*	*FLT3*	
GNA11	*GNAQ*	*GNAS*	*HNF1A*	
HRAS	*IDH1*	*IDH2*	*JAK2*	
JAK3	*KDR*	*KIT*	***KRAS***	
MET	*MLH1*	*MPL*	***MYC***	
NOTCH1	*NPM1*	***NRAS***	*PDGFRA*	
PIK3CA	***PTEN***	*PTPN11*	***PROC***	
RB1	*RET*	*SMAD4*	*SMARCB1*	
SMO	*SRC*	*STK11*	*TERT*	
TP53	*VHL*			

All exons were sequenced in genes in ***bold***.

Panel S1B. 68 gene panel (N=61 samples).

**POINT MUTATIONS**				**AMPLIFICATIONS**	**FUSIONS**	**INDELS**
AKT1	*ALK*	***APC***	***AR***	*AR*	*ALK*	*EGFR* exon 19 deletions
ARAF	***ARID1A***	*ATM*	***BRAF***	*BRAF*	*NTRK1*	*EGFR* exon 20 insertions
BRCA1	***BRCA2***	***CCDN1***	***CCDN2***	*CCNE1*	*RET*	
CCNE1	*CDH1*	***CDK4***	***CDK6***	*CDK4*	*ROS1*	
CDKN2A	***CDKN2B***	*CTNNB1*	***EGFR***	*CDK6*		
ERBB2	*ESR1*	*EZH2*	*FBXW7*	*EGFR*		
FGFR1	***FGFR2***	*FGFR3*	*GATA3*	*ERBB2*		
GNA11	*GNAQ*	*GNAS*	*HNF1A*	*FGFR1*		
HRAS	*IDH1*	*IDH2*	*JAK2*	*FGFR2*		
JAK3	***KIT***	***KRAS***	*MAP2K1*	*KIT*		
MAP2K2	***MET***	*MLH1*	*MPL*	*KRAS*		
MYC	***NF1***	*NFE2L2*	*NOTCH1*	*MET*		
NPM1	***NRAS***	*NTRK1*	***PDGFRA***	*MYC*		
PIK3CA	***PTEN***	*PTPN11*	***RAF1***	*PDGFRA*		
RET	*RHEB*	*RHOA*	*RIT1*	*PIK3CA*		
ROS1	*SMAD4*	*SMO*	*SRC*	*RAF1*		
STK11	*TERT*	***TP53***	*VHL*			

Complete exon coverage for genes in ***bold***.

Panel S1C. 70 gene panel (N=3 samples).

POINT MUTATIONS				AMPLIFICATIONS	FUSIONS	INDELS
AKT1	*ALK**	***APC***	***AR***	*AR*	*ALK*	*EGFR* exon 19 deletions
ARAF	***ARID1A***	*ATM*	***BRAF***	*BRAF*	*FGFR2*	*EGFR* exon 20 insertions
BRCA1	***BRCA2***	***CCND1***	***CCND2***	*CCND1*	*FGFR3*	*ERBB2* exon 19 deletions
CCNE1	*CDH1*	***CDK4***	***CDK6***	*CCND2*	*NTRK1*	*ERBB2* exon 20 insertions
CDKN2A	***CDKN2B***	*CTNNB1*	***EGFR***	*CCNE1*	*RET*	
ERBB2	*ESR1*	*EZH2*	*FBXW7*	*CDK4*	*ROS1*	
FGFR1	***FGFR2****	*FGFR3**	*GATA3*	*CDK6*		
GNA11	*GNAQ*	*GNAS*	*HNF1A*	*EGFR*		
HRAS	*IDH1*	*IDH2*	*JAK2*	*ERBB2*		
JAK3	***KIT***	***KRAS***	*MAP2K1*	*FGFR1*		
MAP2K2	***MET***	*MLH1*	*MPL*	*FGFR2*		
MYC	***NF1***	*NFE2L2*	*NOTCH1*	*KIT*		
NPM1	***NRAS***	*NTRK1**	***PDGFRA***	*KRAS*		
PIK3CA	***PTEN***	*PTPN11*	***RAF1***	*MET*		
RB1	*RET**	*RHEB*	*RHOA*	*MYC*		
RIT1	*ROS1**	*SMAD4*	*SMO*	*PDGFRA*		
SRC	*STK11*	*TERT*	***TP53***	*PIK3CA*		
TSC1	*VHL*			*RAF1*		

Complete exon and partial intron coverage for genes in ***bold***. *Genes with asterisk include rearrangements. *MET* includes exon 14 skipping.

Panel S1D. 73 gene panel (N=26 samples).

POINT MUTATIONS				AMPLIFICATIONS	FUSIONS	INDELS	
AKT1	*ALK*	*APC*	*AR*	*AR*	*ALK*	*APC*	*ARID1A*
ARAF	*ARID1A*	*ATM*	*BRAF*	*BRAF*	*FGFR2*	*ATM*	*BRCA1*
BRCA1	*BRCA2*	*CCND1*	*CCND2*	*CCND1*	*FGFR3*	*BRCA2*	*CDH1*
CCNE1	*CDH1*	*CDK4*	*CDK6*	*CCNE1*	*NTRK1*	*CDKN2A*	*EGFR*
CDKN2A	*CTNNB1*	*DDR2*	*EGFR*	*CDK4*	*RET*	*GATA3*	*KIT*
ERBB2	*ESR1*	*EZH2*	*FBXW7*	*CDK6*	*ROS1*	*MET*	*MLH1*
FGFR1	*FGFR2*	*FGFR3*	*GATA3*	*EGFR*		*MTOR*	*NF1*
GNA11	*GNAQ*	*GNAS*	*HNF1A*	*ERBB2*		*PDGFRA*	*PTEN*
HRAS	*IDH1*	*IDH2*	*JAK2*	*FGFR1*		*RB1*	*SMAD4*
JAK3	*KIT*	*KRAS*	*MAP2K1*	*FGFR2*		*STK11*	*TP53*
MAP2K2	*MAPK1*	*MAPK3*	*MET*	*KIT*		*TSC1*	*VHL*
MLH1	*MPL*	*MTOR*	*MYC*	*KRAS*			
NF1	*NFE2L2*	*NOTCH1*	*NPM1*	*MET*			
NRAS	*NTRK1*	*NTRK3*	*PDGFRA*	*MYC*			
PIK3CA	*PTEN*	*PTPN11*	*RAF1*	*PDGFRA*			
RB1	*RET*	*RHEB*	*RHOA*	*PIK3CA*			
RIT1	*ROS1*	*SMAD4*	*SMO*	*RAF1*			
STK11	*TERT*	*TP53*	*TSC1*				
VHL							

All clinically relevant exons for 73 genes are sequenced. *TERT* includes alterations in the promoter region. *MET* includes exon 14 skipping.

Supplementary Table S2. Next-generation sequencing gene panels for tissue-DNA (N=88).
Panel S2A. 236 gene panel of tissue-DNA (*Foundation Medicine*) (N=22 tissue samples).

**SUBSTITUTIONS, INSERTION/DELETIONS, AND COPY NUMBER ALTERATIONS**								
ABL1	*AKT1*	*AKT2*	*AKT3*	*ALK*	*APC*	*AR*	*ARAF*	*ARFRP1*
ARID1A	*ARID2*	*ASXL1*	*ATM*	*ATR*	*ATRX*	*AURKA*	*AURKB*	*AXL*
BAP1	*BARD1*	*BCL2*	*BCL2L2*	*BCL6*	*BCOR*	*BCORL1*	*BLM*	*BRAF*
BRCA1	*BRCA2*	*BRIP1*	*BTK*	*C11orf30*	*C17orf39*	*CARD11*	*CBFB*	*CBL*
CCND1	*CCND2*	*CCND3*	*CCNE1*	*CD79A*	*CD79B*	*CDC73*	*CDH1*	*CDK12*
CDK4	*CDK6*	*CDK8*	*CDKN1B*	*CDKN2A*	*CDKN2B*	*CDKN2C*	*CEBPA*	*CHEK1*
CHEK2	*CIC*	*CREBBP*	*CRKL*	*CRLF2*	*CSF1R*	*CTCF*	*CTNNA1*	*CTNNB1*
DAXX	*DDR2*	*DNMT3A*	*DOT1L*	*EGFR*	*EP300*	*EPHA3*	*EPHA5*	*EPHB1*
ERBB2	*ERBB3*	*ERBB4*	*ERG*	*ESR1*	*EZH2*	*FAM123B*	*FAM46C*	*FANCA*
FANCC	*FANCD2*	*FANCE*	*FANCF*	*FANCG*	*FANCL*	*FBXW7*	*FGF10*	*FGF14*
FGF19	*FGF23*	*FGF3*	*FGF4*	*FGF6*	*FGFR1*	*FGFR2*	*FGFR3*	*FGFR4*
FLT1	*FLT3*	*FLT4*	*FOXL2*	*GATA1*	*GATA2*	*GATA3*	*GNA11*	*GNA13*
GNAQ	*GNAS*	*GPR124*	*GRIN2A*	*GSK3B*	*HGF*	*HRAS*	*IDH1*	*IDH2*
IGF1R	*IKBKE*	*IKZF1*	*IL7R*	*INHBA*	*IRF4*	*IRS2*	*JAK1*	*JAK2*
JAK3	*JUN*	*KAT6A*	*KDM5A*	*KDM5C*	*KDM6A*	*KDR*	*KEAP1*	*KIT*
KLHL6	*KRAS*	*LRP1B*	*MAP2K1*	*MAP2K2*	*MAP2K4*	*MAP3K1*	*MCL1*	*MDM2*
MDM4	*MED12*	*MEF2B*	*MEN1*	*MET*	*MITF*	*MLH1*	*MLL*	*MLL2*
MPL	*MRE11A*	*MSH2*	*MSH6*	*MTOR*	*MUTYH*	*MYC*	*MYCL1*	*MYCN*
MYD88	*NF1*	*NF2*	*NFE2L2*	*NFKBIA*	*NKX2-1*	*NOTCH1*	*NOTCH2*	*NPM1*
NRAS	*NTRK1*	*NTRK2*	*NTRK3*	*NUP93*	*PAK3*	*PALB2*	*PAX5*	*PBRM1*
PDGFRA	*PDGFRB*	*PDK1*	*PIK3CA*	*PIK3CG*	*PIK3R1*	*PIK3R2*	*PPP2R1A*	*PRDM1*
PRKAR1A	*PRKDC*	*PTCH1*	*PTEN*	*PTPN11*	*RAD50*	*RAD51*	*RAF1*	*RARA*
RB1	*RET*	*RICTOR*	*RNF43*	*RPTOR*	*RUNX1*	*SETD2*	*SF3B1*	*SMAD2*
SMAD4	*SMARCA4*	*SMARCB1*	*SMO*	*SOCS1*	*SOX10*	*SOX2*	*SPEN*	*SPOP*
SRC	*STAG2*	*STAT4*	*STK11*	*SUFU*	*TET2*	*TGFBR2*	*TNFAIP3*	*TNFRSF14*
TOP1	*TP53*	*TSC1*	*TSC2*	*TSHR*	*VHL*	*WISP3*	*WT1*	*XPO1*
ZNF217	*ZNF703*							
**REARRANGEMENTS**								
ALK	*BCL2*	*BCR*	*BRAF*	*EGFR*	*ETV1*	*ETV4*	*ETV5*	*ETV6*
EWSR1	*MLL*	*MYC*	*NTRK1*	*PDGFRA*	*RAF1*	*RARA*	*RET*	*ROS1*
TMPRSS2								

Panel S2B. 315 gene panel of tissue-DNA (*Foundation Medicine, Inc*) (N=52 tissue samples).

**SUBSTITUTIONS, INSERTION/DELETIONS, AND COPY NUMBER ALTERATIONS**								
ABL1	*ABL2*	*ACVR1B*	*AKT1*	*AKT2*	*AKT3*	*ALK*	*AMER1*	*APC*
AR	*ARAF*	*ARFRP1*	*ARID1A*	*ARID1B*	*ARID2*	*ASXL1*	*ATM*	*ATR*
ATRX	*AURKA*	*AURKB*	*AXIN1*	*AXL*	*BAP1*	*BARD1*	*BCL2*	*BCL2L1*
BCL2L2	*BCL6*	*BCOR*	*BCORL1*	*BLM*	*BRAF*	*BRCA1*	*BRCA2*	*BRD4*
BRIP1	*BTG1*	*BTK*	*C11orf30*	*CARD11*	*CBFB*	*CBL*	*CCND1*	*CCND2*
CCND3	*CCNE1*	*CD274*	*CD79A*	*CD79B*	*CDC73*	*CDH1*	*CDK12*	*CDK4*
CDK6	*CDK8*	*CDKN1A*	*CDKN1B*	*CDKN2A*	*CDKN2B*	*CDKN2C*	*CEBPA*	*CHD2*
CHD4	*CHEK1*	*CHEK2*	*CIC*	*CREBBP*	*CRKL*	*CRLF2*	*CSF1R*	*CTCF*
CTNNA1	*CTNNB1*	*CUL3*	*CYLD*	*DAXX*	*DDR2*	*DICER1*	*DNMT3A*	*DOT1L*
EGFR	*EP300*	*EPHA3*	*EPHA5*	*EPHA7*	*EPHB1*	*ERBB2*	*ERBB3*	*ERBB4*
ERG	*ERRFI1*	*ESR1*	*EZH2*	*FAM46C*	*FANCA*	*FANCC*	*FANCD2*	*FANCE*
FANCF	*FANCG*	*FANCL*	*FAS*	*FAT1*	*FBXW7*	*FGF10*	*FGF14*	*FGF19*
FGF23	*FGF3*	*FGF4*	*FGF6*	*FGFR1*	*FGFR2*	*FGFR3*	*FGFR4*	*FH*
FLCN	*FLT1*	*FLT3*	*FLT4*	*FOXL2*	*FOXP1*	*FRS2*	*FUBP1*	*GABRA6*
GATA1	*GATA2*	*GATA3*	*GATA4*	*GATA6*	*GID4*	*GLI1*	*GNA11*	*GNA13*
GNAQ	*GNAS*	*GPR124*	*GRIN2A*	*GRM3*	*GSK3B*	*H3F3A*	*HGF*	*HNF1A*
HRAS	*HSD3B1*	*HSP90AA1*	*IDH1*	*IDH2*	*IGF1R*	*IGF2*	*IKBKE*	*IKZF1*
IL7R	*INHBA*	*INPP4B*	*IRF2*	*IRF4*	*IRS2*	*JAK1*	*JAK2*	*JAK3*
JUN	*KAT6A*	*KDM5A*	*KDM5C*	*KDM6A*	*KDR*	*KEAP1*	*KEL*	*KIT*
KLHL6	*KMT2A*	*KMT2C*	*KMT2D*	*KRAS*	*LMO1*	*LRP1B*	*LYN*	*LZTR1*
MAGI2	*MAP2K1*	*MAP2K2*	*MAP2K4*	*MAP3K1*	*MCL1*	*MDM2*	*MDM4*	*MED12*
MEF2B	*MEN1*	*MET*	*MITF*	*MLH1*	*MPL*	*MRE11A*	*MSH2*	*MSH6*
MTOR	*MUTYH*	*MYC*	*MYCL*	*MYCN*	*MYD88*	*NF1*	*NF2*	*NFE2L2*
NFKBIA	*NKX2-1*	*NOTCH1*	*NOTCH2*	*NOTCH3*	*NPM1*	*NRAS*	*NSD1*	*NTRK1*
NTRK2	*NTRK3*	*NUP93*	*PAK3*	*PALB2*	*PARK2*	*PAX5*	*PBRM1*	*PDCD1LG2*
PDGFRA	*PDGFRB*	*PDK1*	*PIK3C2B*	*PIK3CA*	*PIK3CB*	*PIK3CG*	*PIK3R1*	*PIK3R2*
PLCG2	*PMS2*	*POLD1*	*POLE*	*PPP2R1A*	*PRDM1*	*PREX2*	*PRKAR1A*	*PRKCI*
PRKDC	*PRSS8*	*PTCH1*	*PTEN*	*PTPN11*	*QKI*	*RAC1*	*RAD50*	*RAD51*
RAF1	*RANBP2*	*RARA*	*RB1*	*RBM10*	*RET*	*RICTOR*	*RNF43*	*ROS1*
RPTOR	*RUNX1*	*RUNX1T1*	*SDHA*	*SDHB*	*SDHC*	*SDHD*	*SETD2*	*SF3B1*
SLIT2	*SMAD2*	*SMAD3*	*SMAD4*	*SMARCA4*	*SMARCB1*	*SMO*	*SNCAIP*	*SOCS1*
SOX10	*SOX2*	*SOX9*	*SPEN*	*SPOP*	*SPTA1*	*SRC*	*STAG2*	*STAT3*
STAT4	*STK11*	*SUFU*	*SYK*	*TAF1*	*TBX3*	*TERC*	*TERT**	*TET2*
TGFBR2	*TNFAIP3*	*TNFRSF14*	*TOP1*	*TOP2A*	*TP53*	*TSC1*	*TSC2*	*TSHR*
U2AF1	*VEGFA*	*VHL*	*WISP3*	*WT1*	*XPO1*	*ZBTB2*	*ZNF217*	*ZNF703*
**REARRANGEMENTS**								
ALK	*BCL2*	*BCR*	*BRAF*	*BRCA1*	*BRCA2*	*BRD4*	*EGFR*	*ETV1*
ETV4	*ETV5*	*ETV6*	*FGFR1*	*FGFR2*	*FGFR3*	*KIT*	*MSH2*	*MYB*
MYC	*NOTCH2*	*NTRK1*	*NTRK2*	*PDGFRA*	*RAF1*	*RARA*	*RET*	*ROS1*
TMPRSS2								

**TERT* promoter region

Panel S2C. 324 gene panel of tissue-DNA (*Foundation Medicine, Inc*) (N=5 tissue samples).

**SUBSTITUTIONS, INSERTION/DELETIONS, AND COPY NUMBER ALTERATIONS**								
ABL1	*ACVR1B*	*AKT1*	*AKT2*	*AKT3*	*ALK*	*ALOX12B*	*AMER1*	*APC*
AR	*ARAF*	*ARFRP1*	*ARID1A*	*ASXL1*	*ATM*	*ATR*	*ATRX*	*AURKA*
AURKB	*AXIN1*	*AXL*	*BAP1*	*BARD1*	*BCL2*	*BCL2L1*	*BCL2L2*	*BCL6*
BCOR	*BCORL1*	*BRAF*	*BRCA1*	*BRCA2*	*BRD4*	*BRIP1*	*BTG1*	*BTG2*
BTK	*C11orf30*	*C17orf39*	*CALR*	*CARD11*	*CASP8*	*CBFB*	*CBL*	*CCND1*
CCND2	*CCND3*	*CCNE1*	*CD22*	*CD274*	*CD70*	*CD79A*	*CD79B*	*CDC73*
CDH1	*CDK12*	*CDK4*	*CDK6*	*CDK8*	*CDKN1A*	*CDKN1B*	*CDKN2A*	*CDKN2B*
CDKN2C	*CEBPA*	*CHEK1*	*CHEK2*	*CIC*	*CREBBP*	*CRKL*	*CSF1R*	*CSF3R*
CTCF	*CTNNA1*	*CTNNB1*	*CUL3*	*CUL4A*	*CXCR4*	*CYP17A1*	*DAXX*	*DDR1*
DDR2	*DIS3*	*DNMT3A*	*DOT1L*	*EED*	*EGFR*	*EP300*	*EPHA3*	*EPHB1*
EPHB4	*ERBB2*	*ERBB3*	*ERBB4*	*ERCC4*	*ERG*	*ERRFI1*	*ESR1*	*EZH2*
FAM46C	*FANCA*	*FANCC*	*FANCG*	*FANCL*	*FAS*	*FBXW7*	*FGF10*	*FGF12*
FGF14	*FGF19*	*FGF23*	*FGF3*	*FGF4*	*FGF6*	*FGFR1*	*FGFR2*	*FGFR3*
FGFR4	*FH*	*FLCN*	*FLT1*	*FLT3*	*FOXL2*	*FUBP1*	*GABRA6*	*GATA3*
GATA4	*GATA6*	*GNA11*	*GNA13*	*GNAQ*	*GNAS*	*GRM3*	*GSK3B*	*H3F3A*
HDAC1	*HGF*	*HNF1A*	*HRAS*	*HSD3B1*	*ID3*	*IDH1*	*IDH2*	*IGF1R*
IKBKE	*IKZF1*	*INPP4B*	*IRF2*	*IRF4*	*IRS2*	*JAK1*	*JAK2*	*JAK3*
JUN	*KDM5A*	*KDM5C*	*KDM6A*	*KDR*	*KEAP1*	*KEL*	*KIT*	*KLHL6*
KMT2A	*KMT2D*	*KRAS*	*LTK*	*LYN*	*MAF*	*MAP2K1*	*MAP2K2*	*MAP2K4*
MAP3K1	*MAP3K13*	*MAPK1*	*MCL1*	*MDM2*	*MDM4*	*MED12*	*MEF2B*	*MEN1*
MERTK	*MET*	*MITF*	*MKNK1*	*MLH1*	*MPL*	*MRE11A*	*MSH2*	*MSH3*
MSH6	*MST1R*	*MTAP*	*MTOR*	*MUTYH*	*MYC*	*MYCL*	*MYCN*	*MYD88*
NBN	*NF1*	*NF2*	*NFE2L2*	*NFKBIA*	*NKX2-1*	*NOTCH1*	*NOTCH2*	*NOTCH3*
NPM1	*NRAS*	*NSD3*	*NT5C2*	*NTRK1*	*NTRK2*	*NTRK3*	*P2RY8*	*PALB2*
PARK2	*PARP1*	*PARP2*	*PARP3*	*PAX5*	*PBRM1*	*PDCD1*	*PDCD1LG2*	*PDGFRA*
PDGFRB	*PDK1*	*PIK3C2B*	*PIK3C2G*	*PIK3CA*	*PIK3CB*	*PIK3R1*	*PIM1*	*PMS2*
POLD1	*POLE*	*PPARG*	*PPP2R1A*	*PPP2R2A*	*PRDM1*	*PRKAR1A*	*PRKCI*	*PTCH1*
PTEN	*PTPN11*	*PTPRO*	*QKI*	*RAC1*	*RAD21*	*RAD51*	*RAD51B*	*RAD51C*
RAD51D	*RAD52*	*RAD54L*	*RAF1*	*RARA*	*RB1*	*RBM10*	*REL*	*RET*
RICTOR	*RNF43*	*ROS1*	*RPTOR*	*SDHA*	*SDHB*	*SDHC*	*SDHD*	*SETD2*
SF3B1	*SGK1*	*SMAD2*	*SMAD4*	*SMARCA4*	*SMARCB1*	*SMO*	*SNCAIP*	*SOCS1*
SOX2	*SOX9*	*SPEN*	*SPOP*	*SRC*	*STAG2*	*STAT3*	*STK11*	*SUFU*
SYK	*TBX3*	*TEK*	*TET2*	*TGFBR2*	*TIPARP*	*TNFAIP3*	*TNFRSF14*	*TP53*
TSC1	*TSC2*	*TYRO3*	*U2AF1*	*VEGFA*	*VHL*	*WHSC1*	*WT1*	*XPO1*
XRCC2	*ZNF217*	*ZNF703*						
**REARRANGEMENTS**								
ALK	*BCL2*	*BCR*	*BRAF*	*BRCA1*	*BRCA2*	*CD74*	*EGFR*	*ETV4*
ETV5	*ETV6*	*EWSR1*	*EZR*	*FGFR1*	*FGFR2*	*FGFR3*	*KIT*	*KMT2A*
MSH2	*MYB*	*MYC*	*NOTCH2*	*NTRK1*	*NTRK2*	*NUTM1*	*PDGFRA*	*RAF1*
RARA	*RET*	*ROS1*	*RSPO2*	*SDC4*	*SLC34A2*	*TERC*	*TERT**	*TMPRSS2*

**TERT* promoter region

Panel S2D. 397 gene panel of tissue-DNA (UC San Diego Health Clinical Laboratories) (N=5 tissue samples).

**SINGLE NUCLEOTIDE, SMALL INSERTIONS, OR DELETIONS**									
ABL1	*ABL2*	*ACVR1B*	*AKAP9*	*AKT1*	*AKT2*	*AKT3*	*ALK*	*AMER1*	*APC*
AR	*ARAF*	*ARFRP1*	*ARID1A*	*ARID1B*	*ARID2*	*ASPSCR1*	*ASXL1*	*ATF6*	*ATM*
ATP1A1	*ATP2B3*	*ATR*	*ATRX*	*AURKA*	*AURKB*	*AXIN1*	*AXL*	*BAP1*	*BARD1*
BCL11A	*BCL11B*	*BCL2*	*BCL2L1*	*BCL2L2*	*BCL6*	*BCOR*	*BCORL1*	*BLM*	*BMPR1A*
BRAF	*BRCA1*	*BRCA2*	*BRD4*	*BRIP1*	*BTG1*	*BTK*	*C11orf30*	*CACNA1D*	*CAMTA1*
CARD11	*CASP8*	*CBFB*	*CBL*	*CBLB*	*CCND1*	*CCND2*	*CCND3*	*CCNE1*	*CD274*
CD79A	*CD79B*	*CDC73*	*CDH1*	*CDH11*	*CDK12*	*CDK4*	*CDK6*	*CDK8*	*CDKN1A*
CDKN1B	*CDKN2A*	*CDKN2B*	*CDKN2C*	*CEBPA*	*CHD2*	*CHD4*	*CHEK1*	*CHEK2*	*CIC*
CLTCL1	*COL1A1*	*CREBBP*	*CRKL*	*CRLF2*	*CSF1R*	*CSF3R*	*CTCF*	*CTNNA1*	*CTNNB1*
CUL3	*CYLD*	*DAXX*	*DDIT3*	*DDR2*	*DICER1*	*DNM2*	*DNMT3A*	*DOT1L*	*EBF1*
EGFR	*EIF1AX*	*EP300*	*EPHA3*	*EPHA5*	*EPHA7*	*EPHB1*	*ERBB2*	*ERBB3*	*ERBB4*
ERCC3	*ERCC4*	*ERCC5*	*ERG*	*ERRFI1*	*ESR1*	*ETV1*	*ETV4*	*ETV5*	*ETV6*
EWSR1	*EXT1*	*EZH2*	*FAM46C*	*FANCA*	*FANCC*	*FANCD2*	*FANCE*	*FANCF*	*FANCG*
FANCL	*FAS*	*FAT1*	*FBXO11*	*FBXW7*	*FGF10*	*FGF14*	*FGF19*	*FGF23*	*FGF3*
FGF4	*FGF6*	*FGFR1*	*FGFR2*	*FGFR3*	*FGFR4*	*FH*	*FLCN*	*FLT1*	*FLT3*
FLT4	*FOXA1*	*FOXL2*	*FOXO1*	*FOXP1*	*FRS2*	*FUBP1*	*FUS*	*GABRA6*	*GATA1*
GATA2	*GATA3*	*GATA4*	*GATA6*	*GID4*	*GLI1*	*GMPS*	*GNA11*	*GNA13*	*GNAQ*
GNAS	*GPR124*	*GRIN2A*	*GRM3*	*GSK3B*	*H3F3A*	*HGF*	*HIP1*	*HNF1A*	*HRAS*
HSD3B1	*HSP90AA1*	*IDH1*	*IDH2*	*IGF1R*	*IGF2*	*IKBKE*	*IKZF1*	*IL7R*	*INHBA*
INPP4B	*IRF2*	*IRF4*	*IRS2*	*ITK*	*JAK1*	*JAK2*	*JAK3*	*JUN*	*KAT6A*
KAT6B	*KDM5A*	*KDM5C*	*KDM6A*	*KDR*	*KEAP1*	*KEL*	*KIF5B*	*KIT*	*KLHL6*
KMT2A	*KMT2D*	*KRAS*	*LCP1*	*LIFR*	*LMO1*	*LRIG3*	*LRP1B*	*LYN*	*LZTR1*
MAGI2	*MAML2*	*MAP2K1*	*MAP2K2*	*MAP2K4*	*MAP3K1*	*MCL1*	*MDM2*	*MDM4*	*MED12*
MEF2B	*MEN1*	*MET*	*MITF*	*MLH1*	*MLLT3*	*MLLT4*	*MN1*	*MPL*	*MRE11A*
MSH2	*MSH6*	*MTOR*	*MUTYH*	*MYB*	*MYC*	*MYCL*	*MYCN*	*MYD88*	*MYH11*
MYH9	*NCOA1*	*NCOA2*	*NF1*	*NF2*	*NFE2L2*	*NFKBIA*	*NIN*	*NKX2-1*	*NOTCH1*
NOTCH2	*NOTCH3*	*NPM1*	*NR4A3*	*NRAS*	*NSD1*	*NTRK1*	*NTRK2*	*NTRK3*	*NUMA1*
NUP214	*NUP93*	*NUP98*	*PAK3*	*PALB2*	*PARK2*	*PAX3*	*PAX5*	*PAX7*	*PBRM1*
PCM1	*PDCD1LG2*	*PDGFRA*	*PDGFRB*	*PDK1*	*PIK3C2B*	*PIK3CA*	*PIK3CB*	*PIK3CG*	*PIK3R1*
PIK3R2	*PLCG2*	*PMS1*	*PMS2*	*POLD1*	*POLE*	*PPARG*	*PPP2R1A*	*PRDM1*	*PRDM16*
PREX2	*PRKAR1A*	*PRKCI*	*PRKDC*	*PRSS8*	*PTCH1*	*PTEN*	*PTPN11*	*PTPRC*	*QKI*
RAC1	*RAD21*	*RAD50*	*RAD51*	*RAF1*	*RALGDS*	*RANBP17*	*RANBP2*	*RARA*	*RB1*
RBM10	*RET*	*RICTOR*	*RNF43*	*ROS1*	*RPTOR*	*RUNX1*	*RUNX1T1*	*SDHA*	*SDHB*
SDHC	*SDHD*	*SETBP1*	*SETD2*	*SF3B1*	*SLC34A2*	*SLIT2*	*SMAD2*	*SMAD3*	*SMAD4*
SMARCA4	*SMARCB1*	*SMO*	*SNCAIP*	*SOCS1*	*SOX10*	*SOX2*	*SOX9*	*SPEN*	*SPOP*
SPTA1	*SRC*	*SRGAP3*	*SS18*	*STAG2*	*STAT3*	*STAT4*	*STAT5B*	*STK11*	*SUFU*
SYK	*TAF1*	*TBX3*	*TCF7L2*	*TERC*	*TERT*	*TET2*	*TGFBR2*	*THRAP3*	*TMPRSS2*
TNFAIP3	*TNFRSF14*	*TOP1*	*TOP2A*	*TP53*	*TPR*	*TRIM24*	*TRIM33*	*TRIP11*	*TRRAP*
TSC1	*TSC2*	*TSHR*	*U2AF1*	*VEGFA*	*VHL*	*WHSC1*	*WISP3*	*WRN*	*WT1*
XPO1	*ZBTB2*	*ZMYM2*	*ZNF217*	*ZNF384*	*ZNF521*	*ZNF703*			
**REARRANGEMENTS**									
ALK	*ASPSCR1*	*BRAF*	*BRD4*	*DDIT3*	*EGFR*	*ETV1*	*ETV4*	*ETV5*	*ETV6*
EWSR1	*FGFR1*	*FGFR2*	*FGFR3*	*FOXO1*	*FUS*	*MYB*	*NOTCH2*	*NR4A3*	*NTRK1*
NTRK2	*PDGFRA*	*PPARG*	*RAF1*	*RET*	*ROS1*	*SS18*	*TMPRSS2*		

Supplementary Table S3. Detailed information of invasive glioma patients harboring characterized cfDNA alterations (N=28).

ID	Diagnosis (WHO grade)	Age*	Characterized alterations in cfDNA NGS (%cfDNA)	Prior systemic treatment to blood draw for cfDNA	Characterized alterations in tissue DNA NGS
Both cfDNA and Tissue DNA available (N=18)					
1	GBM (IV)	60	*ATM* R3008H (0.2%)	TMZ+RT	*FGFR3-TACC3* fusion, *TERT* promoter -124C>T
6	GBM (IV)	70	*TP53* D281E (4.2%)	TMZ+RT; antineoplaston therapy	*EGFR* amplification, *PTEN* R159fs*21, *CDKN2A*/B loss, *TERT* promoter -124C>T
14	AA (III)	35	*TP53* Y163C (0.1%)	TMZ+RT; lomustine; bevacizumab+irinotecan	*TP53* P278S, *TP53* R249T, *IDH1* R132S, *ATRX* N1232fs*15
23	AA (III)	52	*TP53* V143M (0.4%)	TMZ+RT	*TP53* P142fs*28, *TP53* S241A, *IDH1* R132H, *ATRX* K1305fs*41
27	GBM (IV)	71	*TP53* Y234C (0.7%)	TMZ+RT	*CSF1R* V32G, *PTEN* I101T
44	GBM (IV)	62	*ATM* R3008H (0.1%)	TMZ+RT	*TERT* promoter -124C>T, *PTEN* loss, *CDKN2C* loss, *CDKN2A*/B loss
50	AO (III)	45	*PDGFRA* R500* (0.62%)	TMZ+RT; lomustine; carboplatin	*NF1* Y2285fs*5, *NF1* F1275fs*8, *NF1* Y1296*, *PIK3CA* Q546R, *PIK3R1* splice site 1743-2A>G, *IDH1* R132H
54	GBM (IV)	70	*TP53* Y220C (3.0%), *TP53* P152L (0.31%)	TMZ+RT	*NF1* loss, *PTEN* loss exons 2-9, *TSC2* loss, *ATR* R1082H, *TP53* R158H, *RB1* loss, *TERT* promoter -124C>T
59	GBM (IV)	70	*TP53* L265P (0.81%)	TMZ+RT; mipsagargin; nilotinib; bevacizumab+lomustine	*BRAF* D594N, *EGFR* A289V, *EGFR* amplification, EGFRvIII, *PIK3CA* G1049R, *CDKN2A*/B loss,
95	GBM (IV)	41	*TP53* S215R (2.34%)	TMZ+RT; bevacizumab+carboplatin; bevacizumab+lomustine	*CDK4* amplification, *PTEN* R130*, *MDM2* amplification, *ARID2* S660*, *TERT* promoter -124C>T
101	GBM (IV)	61	*TP53* V143M (0.2%)	TMZ+RT	*NF1* K938*, *PTEN* N276fs*15
105	AA (III)	25	*IDH1* R132H (2%)	TMZ+RT	*IDH1* R132H, *NOTCH1* F357del, *TP53* E285K, *TP53* splice site 719_782+43del107, *ATRX* D2010fs*1, *FUBP1* Q365*
107	AA (III)	81	*JAK2* V617F (0.3%)	TMZ+RT	*EGFR* amplification, EGFRvIII, *CDKN2A*/B loss, *TERT* promoter-124C>T
111	GBM (IV)	48	*TP53* R249S (0.3%)	TMZ+RT	*EGFR* A289V, *EGFR* amplification, *EGFR* V292L, *PTEN* R130*, *CDKN2A*/B loss, *MTAP* loss exons 2-8, *STAG2* R259*, *TERT* promoter -124C>T
114	OD (II)	43	*GNAS* R201C (0.4%)	No systemic treatment (surgical resection and radiation)	*IDH1* R132H, *ERBB4* R838Q, *TERT* promoter -146C>T
120	GBM (IV)	54	*EGFR* A289V (0.5%), *TP53* S241F (0.2%), *TP53* I254S (0.1%)	TMZ+RT; bevacizumab	*EGFR* A289V, *EGFR* amplification, EGFRvIII, *MDM4* amplification, *CDKN2A*/B loss, *PIK3C2B* amplification

123	GBM (IV)	59	*JAK2* V617F (0.3%), *GNAS* R201H (0.3%)	TMZ+RT; optune+TMZ	*PTEN* Splice site axon 9, *RB1* E675Sfs*16, *TP53* R249S
125	AA (III)	40	*TP53* Y220C (0.9%), *TP53* C238_M243del (0.1%)	TMZ+RT; lomustine+procarbazine+vincristine; bevacizumab+irinotecan	*NF1* F1247fs*18, *NF1* I275fs*14, *PTCH1* G1212S, *IDH1* R132S, *ATRX* T1582fs*24, *TP53* R249G
Only cfDNA available (N=10)					
2	GBM (IV)	61	*MET* amplification**	TMZ+RT; bevacizumab+carboplatin	No tissue NGS results
16	GBM (IV)	45	*JAK2* V617F (0.3%)	TMZ+RT	No tissue NGS results
30	GBM (IV)	56	*TP53* P152S (1.2%)	TMZ+RT	No tissue NGS results
38	GBM (IV)	67	*BRAF* Q257R (0.5%)	TMZ+RT	No tissue NGS results
66	GBM (IV)	47	*BRCA1* Q380* (0.11%)	TMZ+RT; bevacizumab	No tissue NGS results
79	GBM (IV)	66	*TP53* R280I (0.2%)	TMZ+RT; mipsagargin; nilotinib	No tissue NGS results
82	GBM (IV)	54	*TP53* C238S (1.41%)	TMZ+RT; bevacizumab+lomustine	No tissue NGS results
113	GBM (IV)	63	*TP53* E258K (0.2%), *FBXW7* R465H (0.1%)	TMZ+RT; bevacizumab; bevacizumab+lomustine	No tissue NGS results
115	GBM (IV)	59	*TP53* G245S (2.9%), *TP53* R213* (0.3%)	TMZ+RT	No tissue NGS results
128	GBM (IV)	59	*TP53* F134C (0.4%), *TP53* G199E (0.2%), *NF1* G672fs (0.1%)	TMZ+RT	No tissue NGS results

* At the time of blood draw for cfDNA (years).
** %cfDNA is not calculated for genomic amplifications.

Abbreviations: AA, anaplastic astrocytoma; AO, anaplastic oligodendroglioma; cfDNA, cell-free DNA; GBM, glioblastoma multiforme; OD, oligodendroglioma; TMZ+RT, temozolomide with concurrent radiation therapy; %cfDNA, mutant allele frequency.

Supplementary Table S4. Presence of characterized cfDNA alterations and overall survival time from blood draw for cfDNA in invasive glioma patients (N=135).

ID	Diagnosis (WHO grade)	Age*	Characterized cfDNA alterations detected	OS time from blood draw for cfDNA
1	GBM (IV)	60	YES	10.8
2	GBM (IV)	61	YES	11.9
3	GBM (IV)	45	NO	17.3
4	GBM (IV)	57	NO	29.9
5	GBM (IV)	53	NO	9.8
6	GBM (IV)	70	YES	34.8
7	GBM (IV)	81	NO	1.7+
8	GBM (IV)	75	NO	2.7
9	OD (II)	37	NO	16.1+
10	GBM (IV)	87	NO	8.7+
11	GBM (IV)	46	NO	12.5
12	GBM (IV)	31	NO	41.7
13	GBM (IV)	46	NO	6.3
14	AA (III)	35	YES	6.1
15	OD (II)	69	NO	2.1+
16	GBM (IV)	45	YES	7.6
17	GBM (IV)	63	NO	9.6
18	GBM (IV)	78	NO	4.9
19	GBM (IV)	37	NO	28
20	GBM (IV)	58	NO	1.8
21	AA (III)	40	NO	51.6+
22	AA (III)	51	NO	16.6+
23	AA (III)	52	YES	52+
24	GBM (IV)	24	NO	6.5
25	AA (III)	41	NO	52+
26	GBM (IV)	30	NO	7.1
27	GBM (IV)	71	YES	50.3+
28	GBM (IV)	49	NO	10.5
29	GBM (IV)	52	NO	11.3
30	GBM (IV)	56	YES	3.5
31	AA (III)	43	NO	50+
32	GBM (IV)	46	NO	12.5
33	AO (III)	62	NO	4.3
34	GBM (IV)	66	NO	9.2+
35	GBM (IV)	64	NO	5.8
36	GBM (IV)	38	NO	18.5
37	GBM (IV)	56	NO	32.9
38	GBM (IV)	67	YES	2.7
39	AO (III)	42	NO	5.5
40	OD (II)	56	NO	49.9+
41	GBM (IV)	65	NO	18.8
42	AA (III)	31	NO	21.7+
43	GBM (IV)	77	NO	23.4
44	GBM (IV)	62	YES	2.3
45	GBM (IV)	37	NO	6.5
46	GBM (IV)	71	NO	46.9+
47	GBM (IV)	63	NO	15.1
48	GBM (IV)	65	NO	44.4
49	GBM (IV)	53	NO	4.2
50	AO (III)	45	YES	20+
51	GBM (IV)	70	NO	17.7
52	OD (II)	52	NO	14.3+
53	GBM (IV)	48	NO	2.2
54	GBM (IV)	70	YES	11.8
55	GBM (IV)	57	NO	5.2
56	DA (II)	53	NO	16.3
57	AO (III)	53	NO	0.1+
58	GBM (IV)	69	NO	24.4
59	GBM (IV)	70	YES	2.6
60	AA (III)	67	NO	49.5+
61	AA (III)	29	NO	24.2
62	GBM (IV)	64	NO	18.5
63	GBM (IV)	44	NO	15.9
64	OD (II)	61	NO	49+
65	GBM (IV)	82	NO	9.9
66	GBM (IV)	47	YES	16.5
67	OD (II)	66	NO	2.5
68	GBM (IV)	48	NO	47.3+
69	GBM (IV)	71	NO	2.7
70	GBM (IV)	76	NO	35.7
71	DA (II)	29	NO	47.1+
72	DA (II)	31	NO	34.7
73	GBM (IV)	73	NO	27.2+
74	AA (III)	50	NO	15.7
75	AA (III)	54	NO	47.2+
76	AA (III)	34	NO	49.8+
77	GBM (IV)	26	NO	16.1
78	OD (II)	61	NO	16.3
79	GBM (IV)	66	YES	18.1
80	AA (III)	63	NO	49.7+

(Continued on following page)

ID	Diagnosis (WHO grade)	Age*	Characterized cfDNA alterations detected	OS time form blood draw for cfDNA
81	DA (II)	65	NO	29.2
82	GBM (IV)	54	YES	3.8
83	DA (II)	63	NO	17.7
84	GBM (IV)	35	NO	7.3
85	DA (II)	38	NO	19.7
86	GBM (IV)	62	NO	4
87	GBM (IV)	60	NO	5.3
88	GBM (IV)	78	NO	1.9+
89	GBM (IV)	69	NO	27.1
90	GBM (IV)	62	NO	16.7
91	OD (II)	32	NO	50.2+
92	GBM (IV)	58	NO	11.9
93	AA (III)	23	NO	47.4+
94	GBM (IV)	60	NO	5.9
95	GBM (IV)	41	YES	4.5
96	GBM (IV)	45	NO	1.1
97	GBM (IV)	53	NO	8.9
98	OD (II)	45	NO	43+
99	GBM (IV)	66	NO	3.7
100	GBM (IV)	49	NO	45.1+
101	GBM (IV)	61	YES	6.6
102	AA (III)	73	NO	9.7+
103	AA (III)	42	NO	36.1
104	GBM (IV)	63	NO	1.2
105	AA (III)	25	YES	35.5+
106	GBM (IV)	49	NO	14.2+
107	AA (III)	81	YES	9.9
108	GBM (IV)	63	NO	16.6
109	GBM (IV)	47	NO	7.6+
110	GBM (IV)	80	NO	3.9+
111	GBM (IV)	48	YES	4.6+
112	GBM (IV)	61	NO	6.7
113	GBM (IV)	63	YES	0.6
114	OD (II)	43	YES	24.3+
115	GBM (IV)	59	YES	9.1
116	GBM (IV)	62	NO	17.2
117	GBM (IV)	44	NO	2.1
118	GBM (IV)	59	NO	15.9
119	GBM (IV)	53	NO	12.9
120	GBM (IV)	54	YES	7.2
121	GBM (IV)	44	NO	3
122	GBM (IV)	51	NO	18.1
123	GBM (IV)	59	YES	3.8
124	AA (III)	55	NO	3.8
125	AA (III)	40	YES	6.4
126	GBM (IV)	62	NO	8.2
127	DA (II)	55	NO	9
128	GBM (IV)	59	YES	11.5
129	GBM (IV)	60	NO	11.2+
130	GBM (IV)	59	NO	13.4
131	GBM (IV)	71	NO	1.8
132	GBM (IV)	58	NO	9.3
133	GBM (IV)	53	NO	8+
134	GBM (IV)	66	NO	5.2+
135	AA (III)	50	NO	2.9+

* At the time of blood draw for cfDNA (years).

Abbreviations: AA, anaplastic astrocytoma; AO, anaplastic oligodendroglioma; cfDNA, cell-free DNA; GBM, glioblastoma multiforme; OD, oligodendroglioma; WHO, world health organization.

Supplementary Figure S1. Frequency (% of patients) of genomic alterations in tissue-DNA NGS among glioma patients (N=88). Only characterized alterations are shown. A total of 89 different genes were involved (only genes altered in ≥2 samples are shown*).

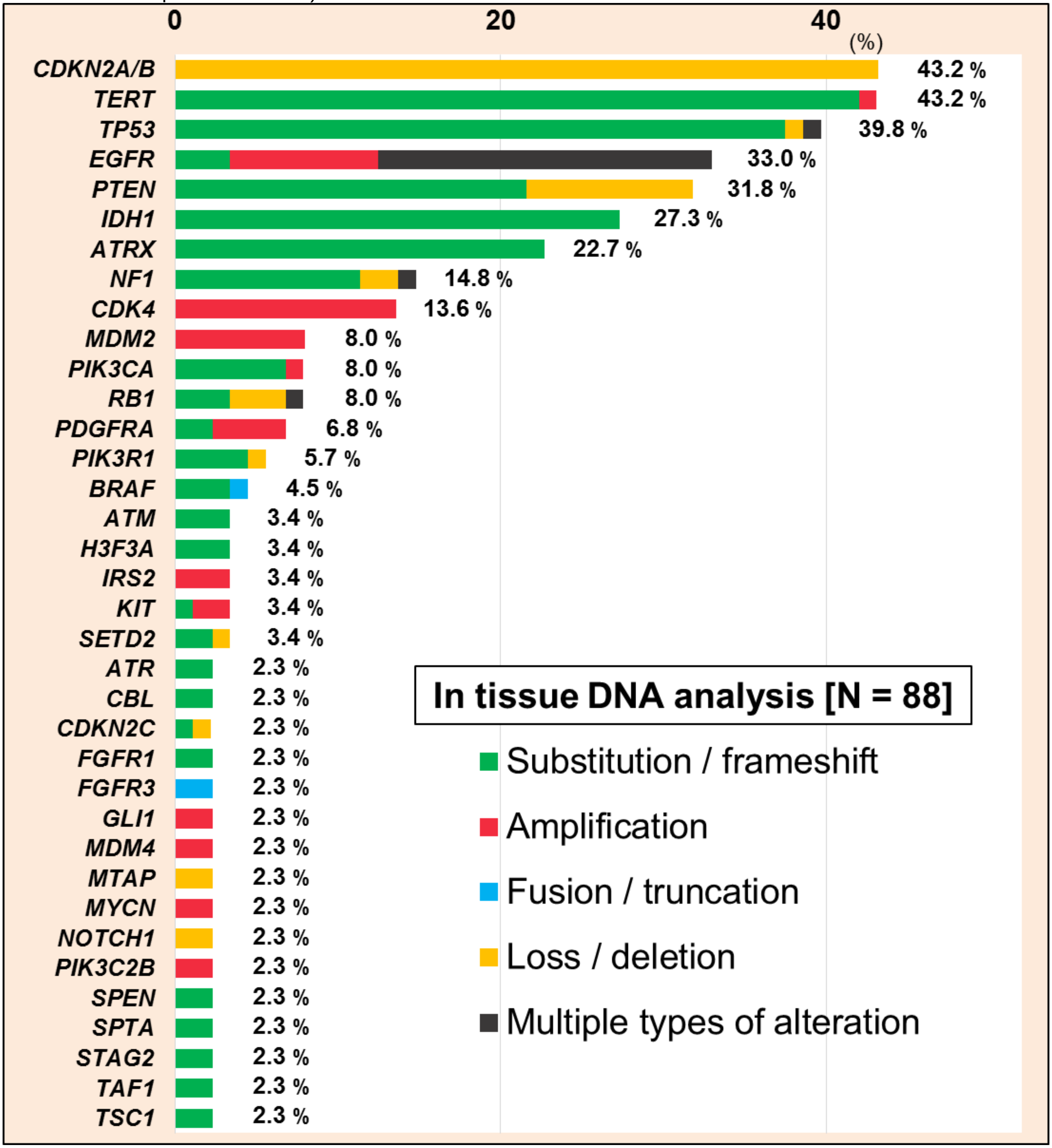

*The following 53 genes were altered in one sample: *ACVR1B, AKT1, AKT3, ARID1A, ARID2, BRIP1, CCNE1, CHD2, CHEK2, CIC, CREBBP, CRKL, CSF1R, CTNNA1, DNMT3A, EP300, ERBB4, FANCA, FANCC, FAS, FAT1, FUBP1, GATA4, HGF, HNF1A, IDH2, JAK2, KDR, KEL, LRP1B, MET, MLL2, MRE11A, MSH2, MUTYH, MYC, NOTCH2, NTRK3, PBRM1, PIK3CG, PLCG2, POLE, PTCH1, PTPN11, QKI, RAD50, SMARCB1, SMO, SOX2, STAT4, TET2, TSC2*, and *WT1.*

Supplementary Figure S2. Kaplan-Meier curve for overall survival (OS) from cfDNA blood test depending on the number of characterized cfDNA alterations amongst patients with brain gliomas (N=135).

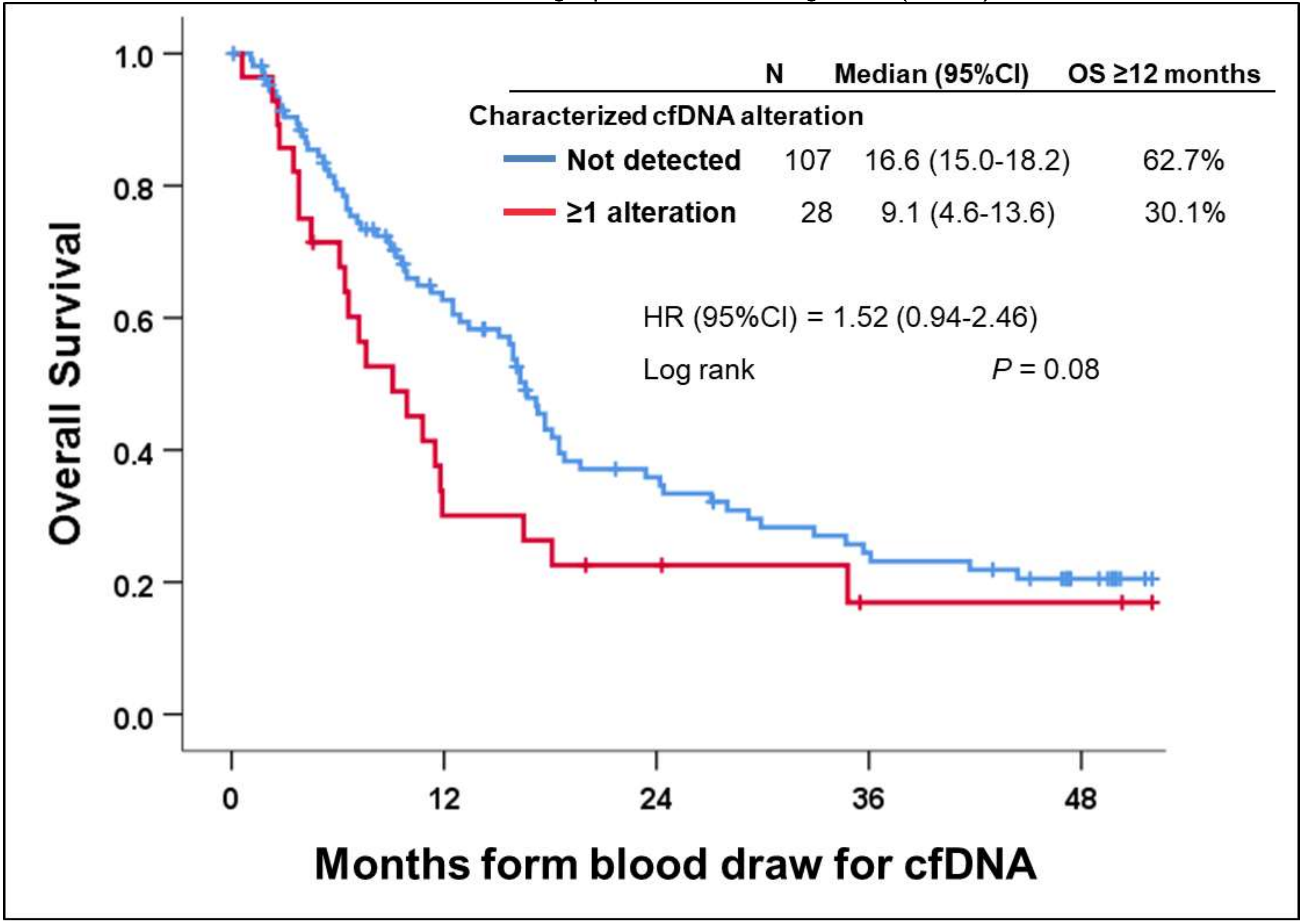

Supplementary Figure S3. Detection rate of characterized cfDNA alterations among GBM patients according to presence of tumor diagnosis (N=93).

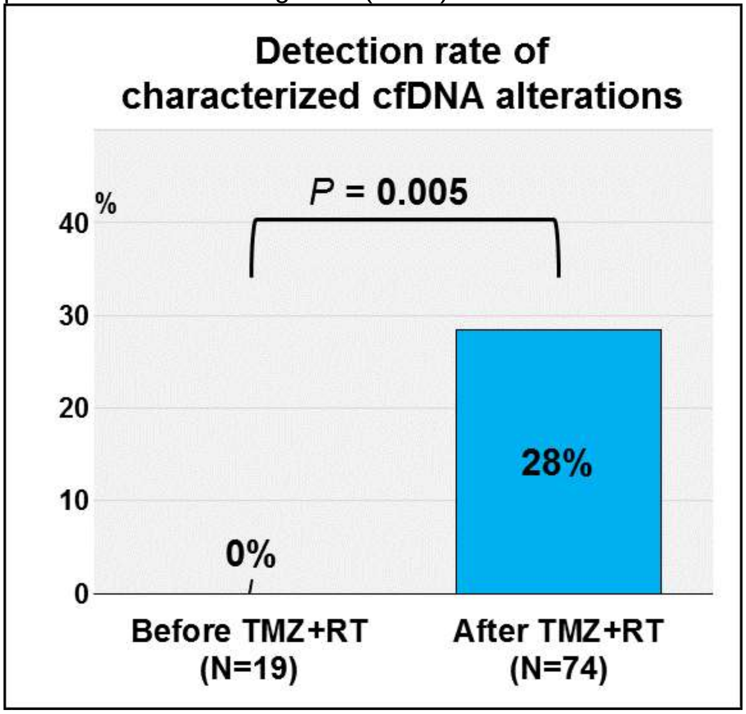

Supplement: Supplementary file 1 [file IJC-148-2839-s001.pdf]
